# De Novo *TRIO* Missense Variants Disrupt Ras‐GEF Domains and Cause Congenital Ventriculomegaly and Hydrocephalus

**DOI:** 10.1155/humu/8870037

**Published:** 2026-04-27

**Authors:** Neel H. Mehta, Evan Dennis, Garrett Allington, Kedous Y. Mekbib, Andrew T. Hale, William C. Davalan, Phan Q. Duy, Emmarose Zilla, Baojian Fan, Ekkehard M. Kasper, Seth L. Alper, Shozeb Haider, Kristopher T. Kahle

**Affiliations:** ^1^ Department of Neurosurgery, Massachusetts General Hospital and Harvard Medical School, Boston, Massachusetts, USA, harvard.edu; ^2^ Department of Neurology, Columbia University Vagelos College of Physicians and Surgeons, New York, New York, USA, vagelos.columbia.edu; ^3^ Department of Neurosurgery, Mayo Clinic Hospital, Rochester, Minnesota, USA, mayoclinic.org; ^4^ Department of Neurosurgery, University of Alabama at Birmingham, Birmingham, Alabama, USA, uab.edu; ^5^ Department of Human Biology and Neuroscience Institute, Univesity of Cape Town, Cape Town, South Africa; ^6^ Department of Neurosurgery, Boston Medical Center, Boston, Massachusetts, USA, bu.edu; ^7^ Faculty of Health Sciences, McMaster University, Hamilton, ON, Canada, mcmaster.ca; ^8^ Division of Nephrology and Vascular Biology Research Center, Beth Israel Deaconess Medical Center, Boston, Massachusetts, USA, bidmc.org; ^9^ Department of Medicine, Harvard Medical School, Boston, Massachusetts, USA, harvard.edu; ^10^ Broad Institute of Harvard and MIT, Cambridge, Massachusetts, USA, broadinstitute.org; ^11^ School of Pharmacy, University College London, London, UK, ucl.ac.uk; ^12^ Division of Genetics and Genomics, Boston Children′s Hospital, Boston, Massachusetts, USA, harvard.edu; ^13^ Harvard Center for Hydrocephalus and Neurodevelopmental Disorders, Massachusetts General Hospital, Boston, Massachusetts, USA, harvard.edu

**Keywords:** congenital, genomics, hydrocephalus, *TRIO*

## Abstract

Congenital hydrocephalus (CH), characterized by congenital ventriculomegaly (CV), affects approximately 0.5–1 per 1000 live births and is a common cause of pediatric neurosurgical intervention, yet its genetic architecture remains incompletely defined. We report a child with syndromic CH requiring cerebrospinal fluid diversion who harbored a pathogenic de novo missense variant in *TRIO* (c.3232C > T; p.(Arg1078Trp)), a gene previously associated with autosomal dominant neurodevelopmental disorders featuring variable head circumference. This case prompted systematic evaluation of *TRIO* variation in our CV/CH cohort (2,697 patient–parent trios) using exome sequencing. We identified five additional unrelated probands with de novo *TRIO* variants, including two novel substitutions affecting the same residue within the Ras‐GEF1 domain (p.(Glu1299Lys) and p.(Glu1299Gly)), yielding significant gene‐level enrichment for protein‐damaging de novo variants (adjusted *p* = 6.12 × 10^−5^). All affected individuals exhibited CV, frequently accompanied by developmental delay and additional structural brain abnormalities. In silico structural modeling predicted that associated variants destabilize critical *TRIO* Ras‐GEF domains required for Rho GTPase activation. Analysis of single‐nucleus transcriptomic data from the developing human neocortex revealed enrichment of *TRIO* expression in multipotent progenitor populations. A systematic literature review identified six additional individuals with *TRIO* de novo variants and reported CV or CH, including an unrelated patient with the same p.(Arg1078Trp) substitution. Together, these findings expand the phenotypic spectrum associated with pathogenic *TRIO* variation to include CV/CH and support *TRIO* as a clinically relevant gene in the genetic evaluation of syndromic CV/CH patients.

## 1. Introduction

Congenital hydrocephalus (CH), often characterized by congenital ventriculomegaly (CV), occurs in approximately 0.5–1 per 1000 live births and is among the most common indications for pediatric neurosurgical intervention and a major contributor to lifelong neurodevelopmental morbidity [1–3]. CH is associated with significant long‐term neurodevelopmental morbidity, with cognitive, motor, or behavioral impairments reported in up to 50% of affected individuals despite treatment [4, 5]. Although CH is often conceptualized as a disorder of cerebrospinal fluid (CSF) circulation and absorption, clinical heterogeneity and variable outcomes after CSF diversion suggest that, in a substantial subset of patients, ventricular enlargement reflects primary disturbances of neurodevelopment rather than a purely secondary hydrodynamic phenomenon [6].

Trio‐based exome sequencing studies have begun to clarify this architecture, demonstrating that rare, damaging de novo variants (DNVs) can underlie syndromic CV/CH while implicating genes involved in neural progenitor biology, differentiation, and tissue morphogenesis [7–11]. These findings support a framework in which genetically encoded perturbations of early brain development can predispose to ventricular enlargement, sometimes culminating in neurosurgically treated hydrocephalus. In this context, identification of recurrent, protein‐damaging DNVs in individual genes has particular value for gene–disease association, phenotype delineation, and clinical genetic evaluation.

*TRIO* encodes a large, multidomain Rho family guanine nucleotide exchange factor (GEF) that regulates Rho GTPase signaling through two catalytic GEF modules and plays established roles in axon guidance, synaptogenesis, and cell adhesion [12–14]. Pathogenic *TRIO* variants cause an autosomal‐dominant *TRIO*‐related neurodevelopmental disorder (TRIO‐NDD), characterized by variable head circumference (microcephaly or macrocephaly), developmental delay/intellectual disability, behavioral phenotypes, and structural brain abnormalities [15–17]. Although ventriculomegaly has been noted in some TRIO‐NDD reports, its relationship to CV or CH requiring CSF diversion has not been systematically evaluated.

Here, we report a child with syndromic, neurosurgically‐treated CH who harbored a pathogenic de novo missense variant in *TRIO*. This observation prompted a gene‐focused interrogation of DNV in *TRIO* across a large CV/CH cohort (> 2,697 patient–parent trios) using exome sequencing. We integrated gene‐level de novo enrichment testing, domain‐level mapping, in silico structural modeling, developmental single‐nucleus transcriptomic profiling, and previously reported cases to systematically evaluate CV and CH as clinical manifestations of *TRIO* variance. Together, these analyses expand the phenotypic spectrum associated with pathogenic *TRIO* variation to include CV/CH and support *TRIO* as a clinically relevant gene in the genetic evaluation of syndromic CV/CH.

## 2. Methods

### 2.1. Cohort Recruitment

This cohort was generated from a combination of CV trios recruited from the Yale Center for Mendelian Genomics and Massachusetts General Hospital, as well as from the GeneDx clinical laboratory referral center. All research procedures adhered to and were conducted under the oversight of the Human Investigation Committee and Human Research Protection Program at both Yale University and Massachusetts General Hospital. Informed written consent was obtained from all participants in alignment with the principles of the Declaration of Helsinki. For GeneDx‐sourced cohort trios, all research methodologies and protocols were conducted in accordance with the Western Institutional Review Board, Puyallup, Washington (WIRB 20162523). For individuals receiving clinical genetic testing through GeneDx (labeled CHYDX), legal guardians of pediatric patients provided written consent for testing. The Western Institutional Review Board waived authorization for use of anonymized, aggregate data in this study. Participants were included in the study based on a diagnosis of CV, which encompassed cases of CH. Samples were collected from patients and available family members (typically patient–parent trios) via buccal swabs (Isohelix SK‐2S DNA kits) or, in a minority of cases, through blood samples. Supporting Information such as clinical records, neuroimaging studies, surgical reports and phenotypic information were gathered when available. Pediatric cases from the GeneDx database were grouped using Human Phenotype Ontology terms. Patients identified as having confirmed, inherited pathogenic variants in genes known to cause syndromes characterized by CH (e.g., L1CAM) were excluded from the cohort. For the control group, unaffected parents and siblings of individuals with autism spectrum disorder were selected from the Simons Simplex Consortium (SSC). The reference population for this analysis included only family members identified as unaffected by the SSC. Access to SSC genomic data was obtained through the National Institute of Mental Health Data Repository, and written informed consent was provided for all SSC participants via the Simons Foundation Autism Research Initiative.

### 2.2. Kinship Analysis

Pedigree relationships between probands and their parents were verified through pairwise identity‐by‐descent (IBD) estimation using PLINK [18]. In all trios, IBD sharing between probands and parents ranged from 45% to 55%, confirming expected parent–offspring relationships. Additional pairwise relatedness among individuals was evaluated using KING [19]. Ancestry assignment in the Yale cohort was carried out by analyzing single‐nucleotide polymorphisms (SNPs) across case and control samples alongside HapMap reference populations, using the EIGENSTRAT method [20]. Kinship evaluation in the GeneDx cohort was completed via a proprietary analysis pipeline incorporating K‐nearest neighbors (KNN) and principal component analysis (PCA).

### 2.3. Exome Sequencing and Variant Calling

Genomic DNA extracted from patient saliva or blood samples underwent exome capture utilizing either the Roche SeqCap EZ MedExome Target Enrichment system or IDT xGen capture technology. Sequencing was performed using paired‐end reads (101 or 148 bp) on Illumina platforms, following previously described protocols. Sequence alignment to the GRCh37/hg19 human reference genome was performed using BWA‐MEM. Variant calling for single‐nucleotide variants and small insertions/deletions employed both GATK HaplotypeCaller and FreeBayes, and subsequent annotation was completed using ANNOVAR [21–23]. Transcript NM_003074.4 and protein isoform NP_003065.3 were used for precise annotation of cDNA and protein changes. Population allele frequency data were annotated using databases including the Exome Aggregation Consortium, gnomAD (Version 2.1.1), and Bravo. Filtering and analysis of variants adhered to GATK best practices and established workflows [21, 24–26]. Missense variant pathogenicity was predicted using MetaSVM and MPC scores. Missense variants were classified as missense damaging (*misD*) when predicted deleterious by MetaSVM or when the MPC score was ≥ 2, whereas missense variants not meeting these criteria were annotated as missense (*mis*). Combined Annotation Dependent Depletion (CADD) scores correspond to CADD v1.3 scaled scores, where higher values indicate greater predicted deleteriousness [27]. Predicted loss‐of‐function (LoF) variants included stop‐gains, stop‐losses, frameshifts, canonical splice site mutations, and start‐losses. Both LoF and D‐mis variants were classified as potentially damaging. Analyses were conducted independently for different variant types, including DNVs, homozygous recessive variants, and rare heterozygous dominant variants, following previously published analytic approaches [28–31]. DNVs in the Yale/MGH cohort were identified in CH trios using the TrioDeNovo pipeline. DNVs in the GeneDx dataset were called using previously described criteria [32–34]. Candidate DNVs were filtered by location (exonic or splice‐site regions), read depth (minimum of 10 reads in both proband and parents), and minor allele frequency (≤4 × 10^−4^ in the GnomAD database) [24]. Further filtering of Yale cohort samples required (i) proband alternate read depth ≥ 5, (ii) alternate allele fraction in the proband ≥ 28*%* when < 10 alternate reads or ≤ 20% when ≥ 10 alternate reads, and (iii) alternate allele frequency in both parents ≤ 3.5*%*.

For the GeneDx cohort, additional criteria were applied: (i) genotype quality (GQ) > 40 across all trio members, (ii) variant quality score log odds (VQSLOD) > −10, (iii) Fisher^′^s exact test Phred − scaled *p* value (FS) < 30, (iv) proband alternate allele count > 4, (v) alternate allele ratio in proband > 0.1, (vi) ratio > 0.15 for variants with equal‐length reference and alternate alleles, (vii) ratio > 0.25 when lengths differ, (viii) ratio < 0.9 for autosomal DNVs, (ix) DNV length < 100 base pairs for both reference and alternate calls, (x) exclusion of variants with VQSLOD < 7 and alternate allele ratio < 0.3, and (xi) exclusion of any variant present in more than two unrelated individuals [21]. Variants passing these filters were visually inspected with internal tools to screen for false positives, which were removed manually. Confirmatory annotations for *TRIO* variants were cross‐checked in the UCSC Genome Browser [35]. Variants in *TRIO* that passed all filters and manual inspection were validated through Sanger sequencing.

### 2.4. De Novo Enrichment Analysis

To evaluate the burden of DNVs in the congenital ventriculomegaly (CV) cohort, the denovolyzeR framework was utilized [36]. The expected number of DNVs per functional category was calculated by summing the mutation probabilities for each class, adjusted by the number of probands multiplied by 2 (to account for diploidy). Observed variant counts were compared with these expectations using a Poisson statistical test, consistent with previously established analytical workflows. Gene‐set enrichment tests were limited to genes with observed or expected variants that fell within defined sets (e.g., those with high brain expression or LoF intolerance). To evaluate enrichment at the individual gene level, expected counts for protein‐altering DNVs were computed using gene‐specific mutation probabilities and cohort size, and observed counts were compared using a Poisson test. Since separate analyses were performed for protein‐altering, protein‐damaging, and LoF DNVs, a Bonferroni correction was applied for multiple comparisons, resulting in a significance threshold of *α* = 8.6 × 10^−7^ (0.05 divided by 3 tests across 19,347 genes).

### 2.5. MutMap Analysis

The locations of previously identified protein‐altering and protein‐damaging DNVs in *TRIO* were mapped as described and localized within a representation of the functional TRIO protein. Domains involved in critical functions as reported in the UniProt database (https://www.uniprot.org/; April 2025) and in literature review [16] were outlined in green. Number of variants represents a running average of all reported variants at each position of the gene, including all pathogenic and likely pathogenetic ClinVar variants (https://www.ncbi.nlm.nih.gov/clinvar; April 2025). Calculation of the running average included reported variants at 10 amino acid residues preceding and following each position.

### 2.6. Single‐Nuclei RNAseq Analysis

Processing and clustering of the single‐nuclei dataset of the developing human neocortex was as previously described [37]. Counts from individual samples were normalized, scaled, and integrated using reciprocal PCA projections. A weighted nearest‐neighbor graph was constructed and used for UMAP embedding. Low‐quality clusters were discarded, and the clustering process was repeated on the filtered dataset. Data were downloaded from the CELLxGENE repository and loaded into Seurat v (5.1) [38]. Average expression across gestational weeks was calculated using the AverageExpression command. The trend line of temporal expression was fitted using the LOESS function with a span of 0.6. Differential expression between cell types was calculated with the FindAllMarkers command, using the Wilcoxon rank sum test. Top *TRIO*‐expressing nuclei were defined as nuclei within the top decile (90th percentile and above) of *TRIO* expression. Gene Ontology analysis of differentially expressed genes in this population was conducted using the package EnrichR (v3.4) with the GO Biological Process 2025 database [39].

### 2.7. In Silico Modeling

The 3097 amino acid sequence of human TRIO protein was from Uniprot (http://www.uniprot.org/), Accession Number O75962. The *TRIO* protein model was built with the AlphaFold3 server (http://www.alphafoldserver.com/). The in silico change in the free energy difference (*Δ* *Δ*G) was calculated using the ICM mutagenesis program (http://www.molsoft.com/) [38]. All figures were generated using the Pymol‐MDAnalysis exploratory project (http://www.pymol.org/pymol).

### 2.8. Systematic Review

Systematic review of all reported *TRIO* gene variants in human cases and animal models was performed in accordance with the Preferred Reporting Items for Systematic Reviews and Meta‐Analyses (PRISMA) guidelines [40]. Comprehensive search of PubMed and Embase was conducted from database inception to March 1, 2025, using a combination of controlled vocabulary and free‐text terms, including “*TRIO*” and “Triple functional domain protein.” Studies were eligible if they (1) described individual human cases with pathogenic or likely pathogenic *TRIO* variants or (2) reported animal models involving manipulation of the *TRIO* gene. For human studies, we included case reports, case series, and cohort studies providing patient‐level genetic and phenotypic data. For animal studies, we included experimental reports characterizing phenotypic, developmental, or behavioral consequences following *TRIO* gene manipulation. We excluded review articles, conference abstracts, studies without available full texts, and studies in which *TRIO* was mentioned without specific patient‐ or model‐level findings. Included articles were also manually screened to any additional relevant publications cited within the manuscript. Although no language restrictions were applied during initial search, final analysis included only articles available in English. Articles were screened first at the title–abstract level, and subsequently at the full‐text level for potential inclusion. Data including variant details (genotype), inheritance pattern, affected protein domain, associated clinical phenotypes, and, where applicable, outcomes from animal models, were then extracted from the final pool of included studies. Extracted data were synthesized narratively and organized into summary Supporting Information tables.

## 3. Results

### 3.1. Identification of a De Novo *TRIO* Variant in a Patient with Neurosurgically‐treated CH

**Figure 1:**
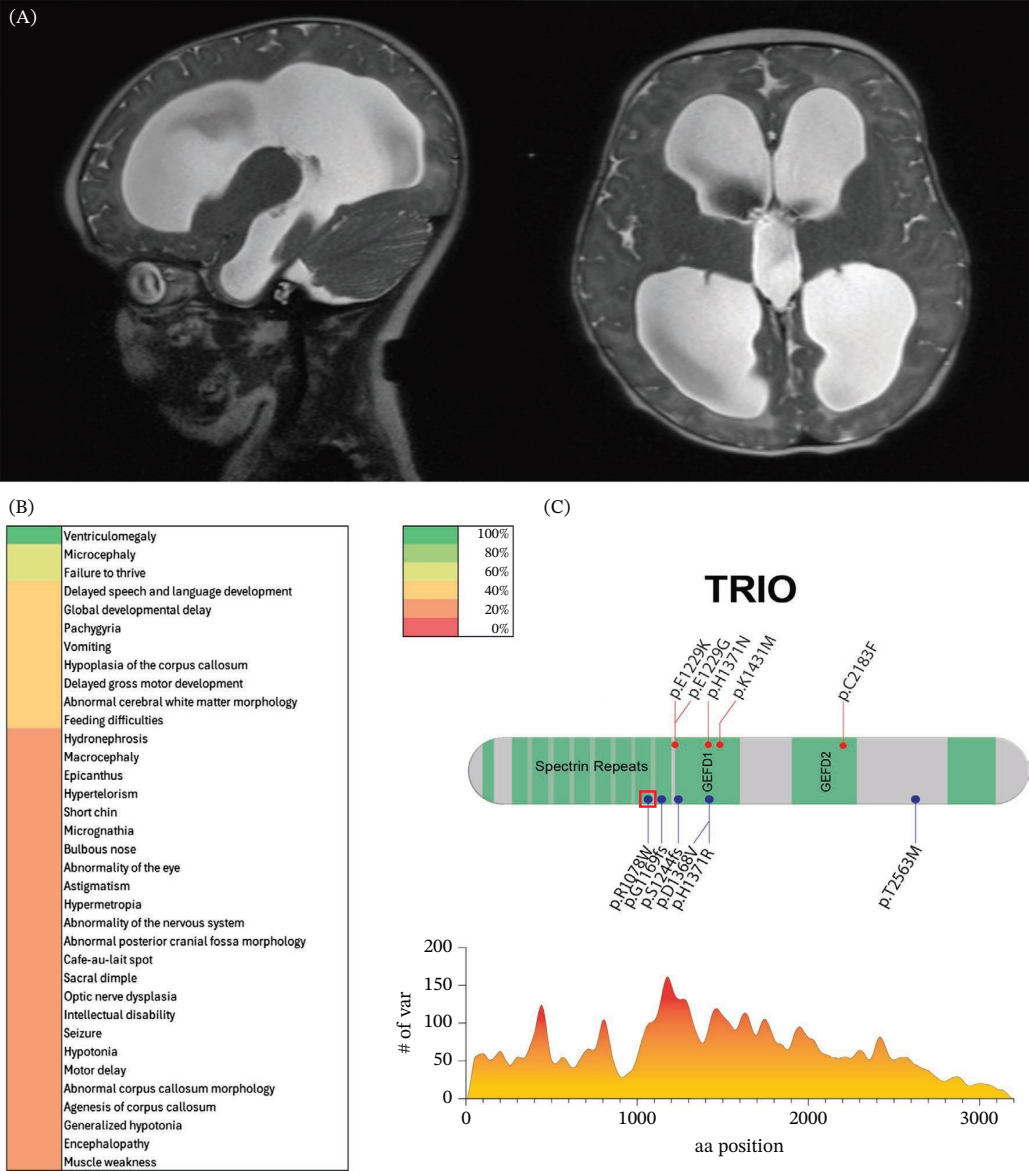
De novo *TRIO* variants cause syndromic congenital ventriculomegaly and hydrocephalus. (A) Brain MRI from the index case demonstrating marked cerebral ventriculomegaly at 4 months of age prior to cerebrospinal fluid diversion. (B) Phenotypic heatmap of probands with de novo *TRIO* variants identified in the congenital ventriculomegaly cohort (*n* = 5). The color scale indicates the occurrence of affected individuals exhibiting each feature. (C) Schematic representation of the *TRIO* protein showing annotated functional domains and the distribution of pathogenic and likely pathogenic missense variants. Variants identified in the CV/CH cohort are shown in red; variants identified through systematic literature review are shown in blue. The p.(Arg1078Trp) variant identified in the index case is highlighted. The lower plot depicts the running average density of reported pathogenic variants across the protein, calculated from ClinVar‐reported variants within a ± 10 amino acid window. Variants are recorded in single‐letter amino acid code.

The index case was a female infant born at term following an uncomplicated pregnancy and perinatal course. Between 2 and 4 months of age she developed progressive macrocephaly with signs of increased intracranial pressure, including vomiting, downgaze preference, and a full fontanelle. Brain MRI demonstrated moderate‐to‐severe CH, and she underwent ventriculoperitoneal shunt placement with subsequent reduction in ventricular size (Figure 1A). Additional neuroimaging findings included hypoplasia of the corpus callosum and mild diffuse cerebral and vermian atrophy. Developmental delays emerged during early childhood. Trio‐based exome sequencing identified a pathogenic de novo missense variant in *TRIO* (NM_003074.4:c.3232C > T; p.(Arg1078Trp)), which was absent in both parents and in population reference databases. No additional pathogenic or likely pathogenic variants relevant to the phenotype were detected. This variant has previously been reported in individuals with TRIO‐NDD and is located within a conserved spectrin repeat domain, suggesting functional relevance. The presence of neurosurgically treated CH in this patient prompted a systematic evaluation of *TRIO* variation in a large cohort of individuals with CV/CH.

### 3.2. Enrichment of De Novo *TRIO* Variants in a Large CV and Hydrocephalus Cohort

We interrogated exome sequencing data from 2,697 proband–parent trios (8,091 exomes) affected by CV, including patients with CH requiring CSF diversion. Variant burden was compared with 1,798 control trios comprising unaffected siblings of individuals with autism spectrum disorder and their unaffected parents from the SSC. DNV enrichment analysis using denovolyzeR demonstrated a marked excess of DNVs in the CV/CH cohort relative to controls (adjusted *p* = 3.03 × 10^−122^), consistent with a substantial contribution of rare DNV to disease risk.

At the gene level, *TRIO* emerged as significantly enriched for protein‐altering DNV in the cohort (five independent events; adjusted *p* = 6.12 × 10^−5^; Table 1), exceeding expectations based on gene‐specific mutation rates. Four variants were classified as protein‐damaging missense substitutions (MetaSVM deleterious and/or MPC ≥ 2), and one additional variant was protein‐altering. *TRIO* is highly constrained for missense variation (gnomAD missense *Z* − score = 4.69), and no de novo *TRIO* variants were observed in the control cohort. The probability of observing five protein‐altering DNV in *TRIO* by chance in a cohort of this size was 1.14 × 10^−5^, supporting a nonrandom association.

**Table 1:** De novo variants in *TRIO* identified in a large cohort (> 2697) of proband–parent trios with congenital ventriculomegaly.

Patient ID	Chromosome	Position	Ref allele	Alt allele	Gene	Variant class	AA change	GnomAD MAF	CADD	MPC
9407016	5	14388735	G	A	*TRIO*	*misD*	p.(Glu1299Lys)	—	34	2.0711495
6755562	5	14388736	A	G	*TRIO*	*misD*	p.(Glu1299Gly)	—	33	2.1058088
2053950	5	14390392	C	A	*TRIO*	*misD*	p.(His1371Asn)	—	33	2.0407752
8449411	5	14394220	A	T	*TRIO*	*misD*	p.(Lys1431Met)	—	32	2.2356751
4149673	5	14482773	G	T	*TRIO*	*mis*	p.(Cys2183Phe)	4.09E‐06	16.78	0.4605982

*Note:* Variant class: *misD*, missense variant predicted to be damaging (MetaSVM deleterious or MPC ≥ 2); *mis*, missense variant not meeting damaging criteria. *CADD*, Combined Annotation Dependent Depletion v1.3 scaled score.

Abbreviations: *pLI*, probability of loss‐of‐function intolerance; *gnomAD MAF*, minor allele frequency in the gnomAD database; *CADD*, Combined Annotation Dependent Depletion; *MPC*, missense badness–PolyPhen‐2–constraint score.

### 3.3. Clinical Features of Patients With De Novo *TRIO* Variants

All five probands with de novo *TRIO* variants identified in our cohort exhibited CV (Figure 1B). As IRB approvals and data‐sharing agreements restricted reporting of individual‐level management details for nonindex cases, the frequency of neurosurgical intervention could not be reported beyond the index case. In addition to ventricular enlargement, affected individuals frequently demonstrated syndromic features. Developmental delay was observed in multiple domains, including global developmental delay, speech and language delay, and gross motor delay. Structural brain abnormalities beyond ventriculomegaly were common and included hypoplasia of the corpus callosum, pachygyria, and abnormal cerebral white matter morphology. Head circumference varied across individuals, consistent with the known phenotypic heterogeneity of TRIO‐NDD.

### 3.4. De Novo *TRIO* Variant Cluster Within and Destabilize Ras‐GEF Domains

To assess potential functional consequences of *TRIO* variants identified in patients with CV/CH, we mapped all DNV from the index case and cohort analysis onto the annotated *TRIO* protein (Figures 1C and S1). *TRIO* encodes a large, multidomain protein comprising a SEC14 lipid–binding domain, multiple spectrin repeats, two catalytic GEF modules (GEFD1 and GEFD2), Src homology 3 (SH3) domains, and an immunoglobulin‐like domain. Notably, four of the five newly identified protein‐damaging DNVs localized to the DH1 subdomain of the Ras‐GEF1 catalytic region (Amino Acids 1292–1467), a critical domain responsible for Rac1 activation. The remaining missense variant localized to the PH2 subdomain of the GEF2 region.

**Figure 2:**
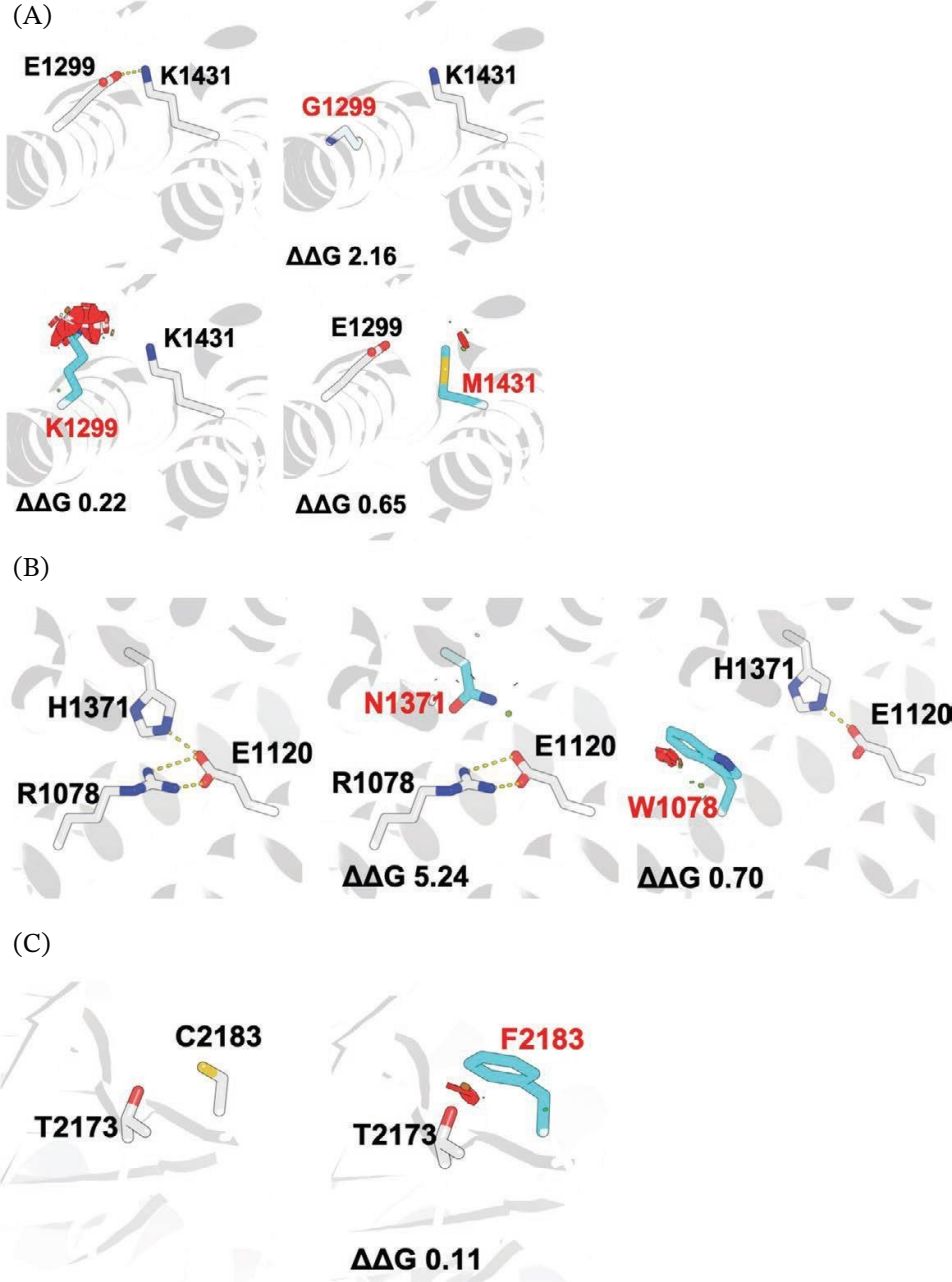
Structural modeling of de novo *TRIO* variants predicts destabilization of Ras‐GEF domains. (A) AlphaFold3‐based structural modeling of the Ras‐GEF1 (DH1) domain illustrating disruption of the stabilizing ion‐pair interaction between residues Glu1299 and Lys1431 by the p.(Glu1299Gly), p.(Glu1299Lys), and p.(Lys1431Met) variants. Residues and variants are written in single‐letter amino acid coding. (B) Structural modeling of the spectrin‐domain variant p.(Arg1078Trp) and the previously reported p.His1371Asn variant, demonstrating disruption of conserved electrostatic and hydrogen‐bonding interactions at the interface between the immunoglobulin‐like and pleckstrin homology domains. Residues and variants are written in single‐letter amino acid coding. (C) Modeling of the Ras‐GEF2 (DH2) domain variant p.(Cys2183Phe) showing introduction of steric clashes and restriction of local backbone flexibility. Predicted free‐energy changes (*Δ* *Δ*G) are indicated for each variant. Residues and variants are written in single‐letter amino acid coding.

The substitutions affecting Glu1299 (p.(Glu1299Gly) and p.(Glu1299Lys)) disrupt a conserved ion‐pair interaction between Glu1299 and Lys1431 that stabilizes adjacent *α*‐helices within the DH1 domain (Figure 2A). In silico modeling predicted that p.(Glu1299Gly) abolishes this interaction by losing the negatively charged side chain, resulting in a destabilizing free‐energy change (*Δ* *Δ*G = 2.16 kcal/mol). The p.(Glu1299Lys) substitution converts this stabilizing interaction into electrostatic repulsion and introduces local steric clashes (*Δ* *Δ*G = 0.22 kcal/mol). Similarly, the p.(Lys1431Met) variant disrupts the same ion‐pair interaction (*Δ* *Δ*G = 0.65 kcal/mol). Variants within this region have been shown previously to impair Rac1 activation, consistent with a LoF mechanism affecting Ras‐GEF1 catalytic activity.

The index case variant p.(Arg1078Trp), previously reported in unrelated individuals [16, 41], localizes to the seventh spectrin repeat and participates in a hydrogen‐bonding network stabilizing the interface between the immunoglobulin‐like (IGH‐1) and pleckstrin homology (PH1) domains (Figure 2B). Modeling predicted that substitution of arginine with the bulky, hydrophobic tryptophan residue disrupts local electrostatic interactions and introduces steric strain, resulting in an unfavorable free‐energy change (*Δ* *Δ*G = 0.70 kcal/mol). A variant affecting the same interaction network (p.(His1371Asn)) produced a marked destabilizing effect (*Δ* *Δ*G = 5.24 kcal/mol), supporting the functional importance of this interface.

Finally, the p.(Cys2183Phe) variant localized to the DH2 subdomain of the GEF2 region. Structural modeling indicated that replacement of cysteine with phenylalanine introduces steric clashes with adjacent residues, restricting local backbone flexibility and mildly destabilizing the domain (*Δ* *Δ*G = 0.11 kcal/mol) (Figure 2C). Although less disruptive than variants in the Ras‐GEF1 region, this alteration may further impair Rho GTPase signaling.

Collectively, these analyses demonstrate that CV/CH‐associated de novo *TRIO* variants cluster within functionally critical Ras‐GEF domains or likely destabilize conserved structural interfaces, supporting a shared pathogenic mechanism involving impaired Rho GTPase activation.

### 3.5. *TRIO* Is Enriched Across Multiple Progenitor‐Derived Neural Populations During Human Cortical Development

**Figure 3:**
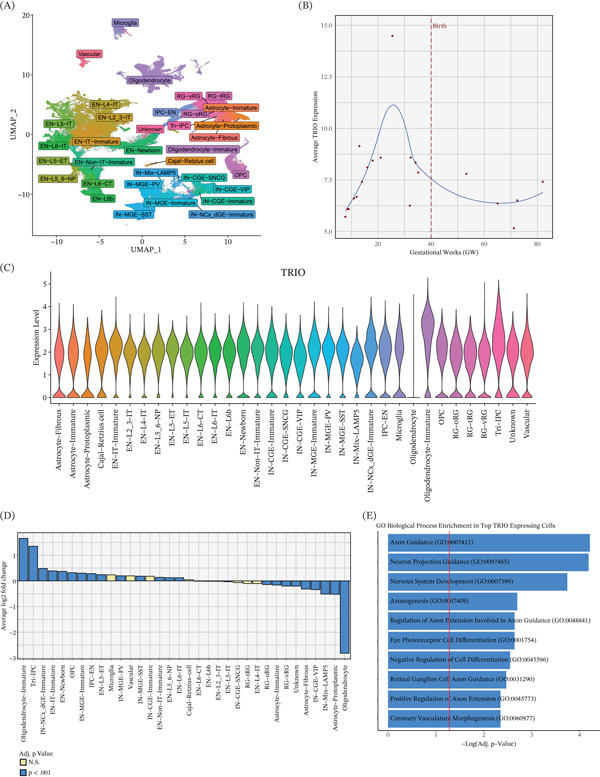
*TRIO* expression in the developing human neocortex. (A) UMAP visualization of 232,328 nuclei from a single‐nucleus RNA sequencing atlas of the developing human neocortex, colored by cell type. (B) Temporal expression of *TRIO* across gestational development and early postnatal stages; the vertical line denotes birth. (C) Violin plots showing *TRIO* expression across annotated neural and glial cell populations. (D) Differential expression of *TRIO* across cell types relative to all other nuclei, highlighting enrichment in progenitor‐derived neural populations. (E) Gene Ontology biological process enrichment for genes coexpressed with *TRIO* in the top decile of *TRIO*‐expressing nuclei. The red vertical line indicates the threshold for Benjamini–Hochberg adjusted statistical significance.

To contextualize the developmental relevance of *TRIO* variants associated with CV/CH, we examined *TRIO* expression in a single‐nucleus RNA sequencing atlas of the developing human neocortex comprising 232,328 nuclei across gestational and early postnatal stages (Figure 3A). *TRIO* expression was highest during midgestation, peaking around gestational Week 25, followed by gradual decline after birth (Figure 3B). Across 34 annotated neural and glial cell populations, *TRIO* showed broad expression but was significantly enriched in specific progenitor‐derived developmental populations (Figure 3C), including tripotential intermediate progenitor cell–derived lineages (Figure 3D). Tri‐IPCs represent a multipotent progenitor population capable of generating neuronal, astrocytic, and oligodendroglial lineages. Gene Ontology analysis of genes coexpressed with *TRIO* in the top decile of *TRIO*‐expressing nuclei demonstrated significant enrichment for biological processes related to nervous system development, axon guidance, and neuron projection guidance (Figure 3E). These data support a role for *TRIO* in progenitor cell populations during critical periods of cortical development and provide a biological context for the association of pathogenic *TRIO* variants with CV [8, 42, 43].

### 3.6. Previously Reported *TRIO* Variants and Animal Models Support a Conserved Role in Brain and Ventricular Development

Our systematic literature search yielded 200 articles from PubMed, Embase, and Web of Science. The title and abstract screening process resulted in 98 articles reviewed in full text, 51 of which met the eligibility criteria for the final analysis (Figure S2). A total of 17 human studies [15–17, 41, 44–56] reported pathogenic or likely pathogenic *TRIO* variants in 91 patients (Table S1, S2). *TRIO* protein variants were found across functional domains, including the SEC14 domain (*n* = 1), spectrin repeat regions (*n* = 36), DH1 (*n* = 4), GEF1 (*n* = 24), and GEF2 (*n* = 3) domains. Most variants occurred de novo (*n* = 51), although instances of inherited pathogenic variants were also identified (*n* = 20). *TRIO* variants in six patients with reported CV or CH involved de novo spectrin domain variant c.3232C > T (p.(Arg1078Trp)) [15], de novo GEF1 domain variant c.4112A > G (p.(His1371Arg)) [15], and DNVs c.3727del (p.(Ser1244Leufs∗23)) [15], c.3506delG (p.(Gly1169AlafsTer11)) [50], c.4103A > T (p.(Aps1368Val)), and c.7688C > T (p.(Thr2563Met)) [54]. Neurodevelopmental abnormalities represented the most frequently observed clinical phenotypes, including developmental delay, intellectual disability, attention deficit hyperactivity disorder, and autism spectrum disorder. Several cases also presented with structural brain anomalies, such as Chiari malformation [15, 41] and agenesis of the corpus callosum [15, 46].

A comprehensive review identified more than 31 animal models in which the *TRIO* gene is disrupted, highlighting the fundamental role of TRIO signaling in neural development, cytoskeletal organization, and craniofacial morphogenesis across species (Table S3). Complete *TRIO* knockout in mice (*n* = 11) caused embryonic or perinatal lethality with severe brain malformations, whereas heterozygotes showed reduced brain size and behavioral deficits (Table S3). The forebrain‐specific Rac1 conditional knockout mouse (*Foxg1-Cre; Rac1^flox/flox^*) [57] exhibited enlarged lateral ventricles. This model selectively deleted Rac1, a Rho GTPase downstream of TRIO, in telencephalic neural progenitors beginning at embryonic Day 9.5, resulting in a significantly reduced size of the cerebral cortex and striatum, accompanied by a marked enlargement of the lateral ventricles. Additional identified *TRIO* models exhibiting broad neurological phenotypes included rats (*n* = 1), zebrafish (*n* = 2), *Xenopus* (*n* = 3), *Drosophila* (*n* = 12), *Caenorhabditis elegans* (*n* = 1), and chick (*n* = 1), further characterized in Table S3. These models collectively confirm TRIO′s domain‐specific, evolutionarily conserved role in brain development and disease.

## 4. Discussion

In this study, we identify de novo missense variants in *TRIO* as a genetic contributor to a syndromic form of CV and CH. Across a large trio‐based CV/CH cohort, we observed a significant enrichment of protein‐damaging de novo *TRIO* variants, including substitutions affecting a conserved residue within the Ras‐GEF1 catalytic domain. Integration of gene‐level enrichment, domain‐level clustering, structural modeling, developmental transcriptomic data, and systematic literature review supports *TRIO* variants increasing CV and CH risk, and highlights this phenotype as a recurrent clinical feature of TRIO‐NDD.

Pathogenic variants in *TRIO* are a recognized cause of autosomal dominant TRIO‐NDD, which is characterized by developmental delay, intellectual disability, behavioral abnormalities, and variable head circumference [44, 47, 50]. Prior studies have emphasized the role of domain‐specific effects in shaping phenotypic variability, with gain‐of‐function missense variants—often clustering in spectrin repeat regions—associated with macrocephaly [16] and LoF or Ras‐GEF1–disrupting variants associated with microcephaly [58]. Our findings extend this genotype–phenotype framework by demonstrating that both spectrin‐domain and Ras‐GEF1–domain DNVs can be associated with CV or hydrocephalus.

Several lines of evidence support the pathogenic relevance of the *TRIO* variants identified in this cohort. First, *TRIO* demonstrated significant gene‐level enrichment for protein‐altering DNVs relative to expectation, with no such variants observed in control trios. Second, affected residues clustered within functionally critical domains, particularly the Ras‐GEF1 catalytic region, and recurrently disrupted conserved structural interactions. Third, in silico modeling consistently predicted destabilization of catalytic or interdomain interfaces essential for Rho GTPase activation. Finally, *TRIO* expression was enriched in multipotent progenitor populations during midgestational cortical development, providing a biological context for the observed neurodevelopmental phenotypes.

The mechanisms by which pathogenic *TRIO* variants contribute to ventricular enlargement are likely multifactorial. *TRIO* functions as a GEF factor for Rac1, a key regulator of cytoskeletal dynamics, cell migration, and progenitor differentiation [16, 59–62]. Disruption of Rac1 signaling has been implicated in abnormal neural progenitor behavior, impaired tissue morphogenesis, and ventricular enlargement in animal models [63–65]. Consistent with this, several genes previously implicated in syndromic CV/CH (including *TRIM71*, *SMARCC1*, *PTEN*, *PIK3CA*, and *LDB1*) converge on pathways governing neural progenitor proliferation, differentiation, and structural organization [31, 34, 66]. Our findings further support a model in which CV in patients with pathogenic *TRIO* variants may reflect genetically encoded perturbations of early brain development, rather than isolated abnormalities of CSF hydrodynamics [42].

From a clinical genetics perspective, these results have several implications. First, they expand the phenotypic spectrum associated with pathogenic *TRIO* variation to include CV/CH, reinforcing the importance of systematic advanced brain imaging review in individuals diagnosed with TRIO‐NDD. Second, they support the inclusion of *TRIO* in gene lists and interpretive frameworks used to evaluate patients with syndromic ventriculomegaly or hydrocephalus, particularly when accompanied by developmental delay or additional structural brain abnormalities. Finally, these findings highlight the potential utility of trio‐based exome sequencing in selected patients with CV/CH, for whom identification of an underlying genetic etiology may inform prognosis, counseling, and longitudinal management [67, 68].

This study has several limitations. Although our cohort represents the largest trio‐based CV/CH dataset assembled to date, our analyses focused primarily on de novo coding variation and did not systematically evaluate inherited, noncoding, or epigenetic mechanisms that may also contribute to disease risk. In addition, although structural modeling and transcriptomic analyses provide supportive biological context, functional validation of individual *TRIO* variants in experimental systems was beyond the scope of this study. Future studies incorporating variant‐specific cellular or animal models will be important for elucidating precise mechanistic links between disrupted *TRIO* signaling and ventricular morphogenesis. Another limitation relates to phenotypic ascertainment. Ventriculomegaly may be underreported in published TRIO‐NDD cohorts, potentially leading to underestimation of its true prevalence among affected individuals. Finally, because this cohort was assembled through multiple institutions and diagnostic laboratories, our IRB approvals and data‐sharing agreements restrict access to individual‐level clinical and imaging data. Consequently, detailed genotype–phenotype correlations could not be presented in the current analysis. Future studies integrating systematic neuroimaging with individual‐level genetic and clinical data will be important to better define the neuroradiographic spectrum of *TRIO*‐associated ventriculomegaly and hydrocephalus and to clarify its implications for neurosurgical management.

## 5. Conclusions

We identify de novo missense variants in *TRIO* as a genetic contributor to syndromic CV/CH. Protein‐damaging variants cluster within functionally critical Ras‐GEF domains and are significantly enriched in a large trio‐based CV/CH cohort. These findings expand the phenotypic spectrum associated with pathogenic *TRIO* variation and support inclusion of *TRIO* in the genetic evaluation of patients with syndromic CV/CH.

## Author Contributions

Conceptualization: N.H.M., E.D., G.A., K.Y.M., W.C.D., and K.T.K. Data curation: N.H.M., E.D., G.A., K.Y.M., W.C.D., and B.F. Formal analysis: N.H.M., E.D., G.A., K.Y.M., W.C.D., and BF. Funding acquisition: K.T.K. Investigation: N.H.M., E.D., G.A., K.Y.M., W.C.D., and K.T.K. Methodology: N.H.M., E.D., G.A., K.Y.M., W.D., and K.T.K. Writing—original draft: N.H.M., E.D., G.A., K.Y.M., A.T.H., W.C.D., and K.T.K. Writing—review and editing: N.H.M., E.D., G.A., K.Y.M., A.T.H., W.C.D., P.Q.D., E.Z., B.F., S.L.A., S.H., and K.T.K.

## Funding

This study was supported by the National Institutes of Health (10.13039/100000002) (R01NS109358, R01NS111029, R01NS117609).

## Conflicts of Interest

The authors declare no conflicts of interests.

## Supporting information


**Supporting Information** Additional supporting information can be found online in the Supporting Information section. Figures S1–S2 and Tables S1–S3.

## Data Availability

Any data presented in this manuscript can be obtained upon reasonable request to the corresponding author: kahle.kristopher@mgh.harvard.edu.
