# Supplementary material for: De Novo TRIO Missense Variants Disrupt Ras‐GEF Domains and Cause Congenital Ventriculomegaly and Hydrocephalus
Source: Hum Mutat. 2026 Apr 27;2026:8870037. doi: 10.1155/humu/8870037 (PMC13115915; doi:10.1155/humu/8870037)
Supplement: Supplementary file 1 — Supporting Information Additional supporting information can be found online in the Supporting Information section. Figures S1–S2 and Tables S1–S3. [file HUMU-2026-8870037-s001.docx]

**Supplemental Figure 1. Modeling of the human *TRIO* gene.** A) The human TRIO model generated by AlphaFold3, illustrating high pIDDT scores for the structured core regions. B) The sites of mutations are all present in the structured core region; C) The predicted aligned error (PAE) plot indicates well defined relative position and orientations for the mutants. Relevant residues are written in single-letter amino acid code.

**
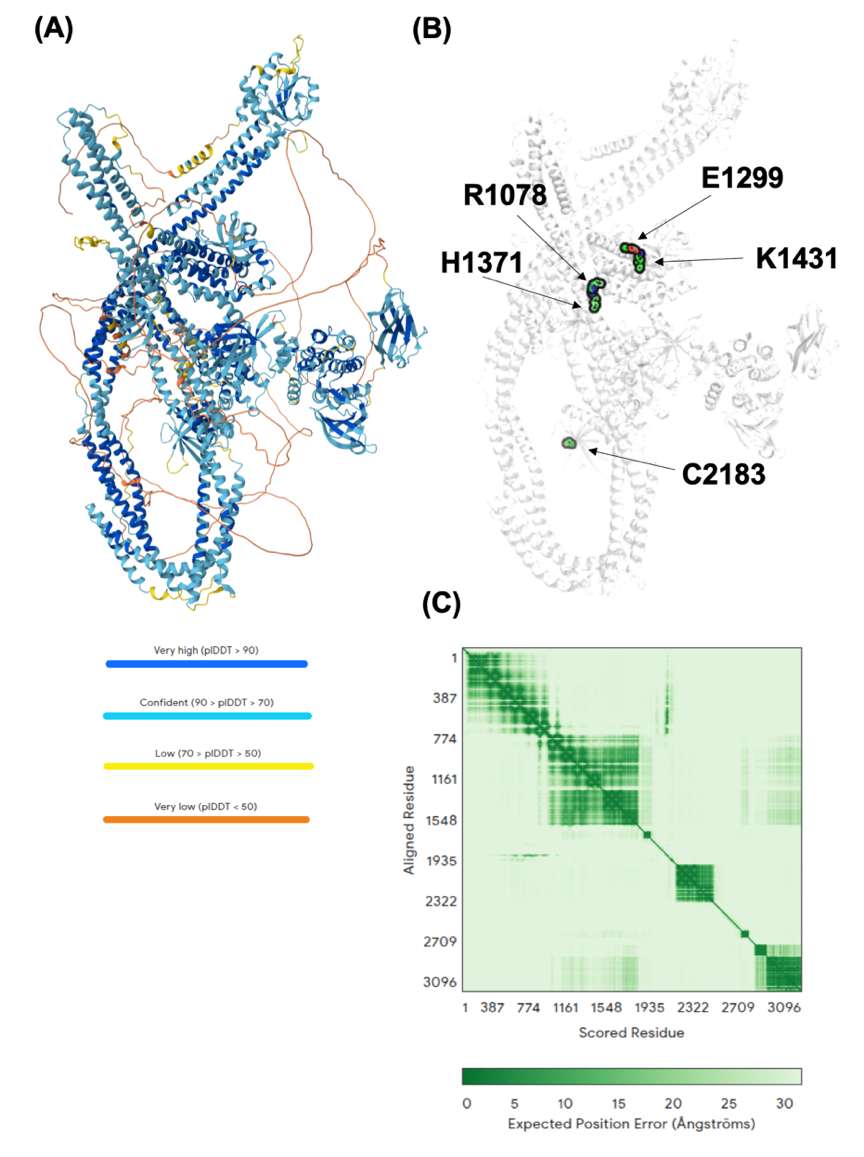
**

**Supplemental Figure 2.** PRISMA flow diagram

**
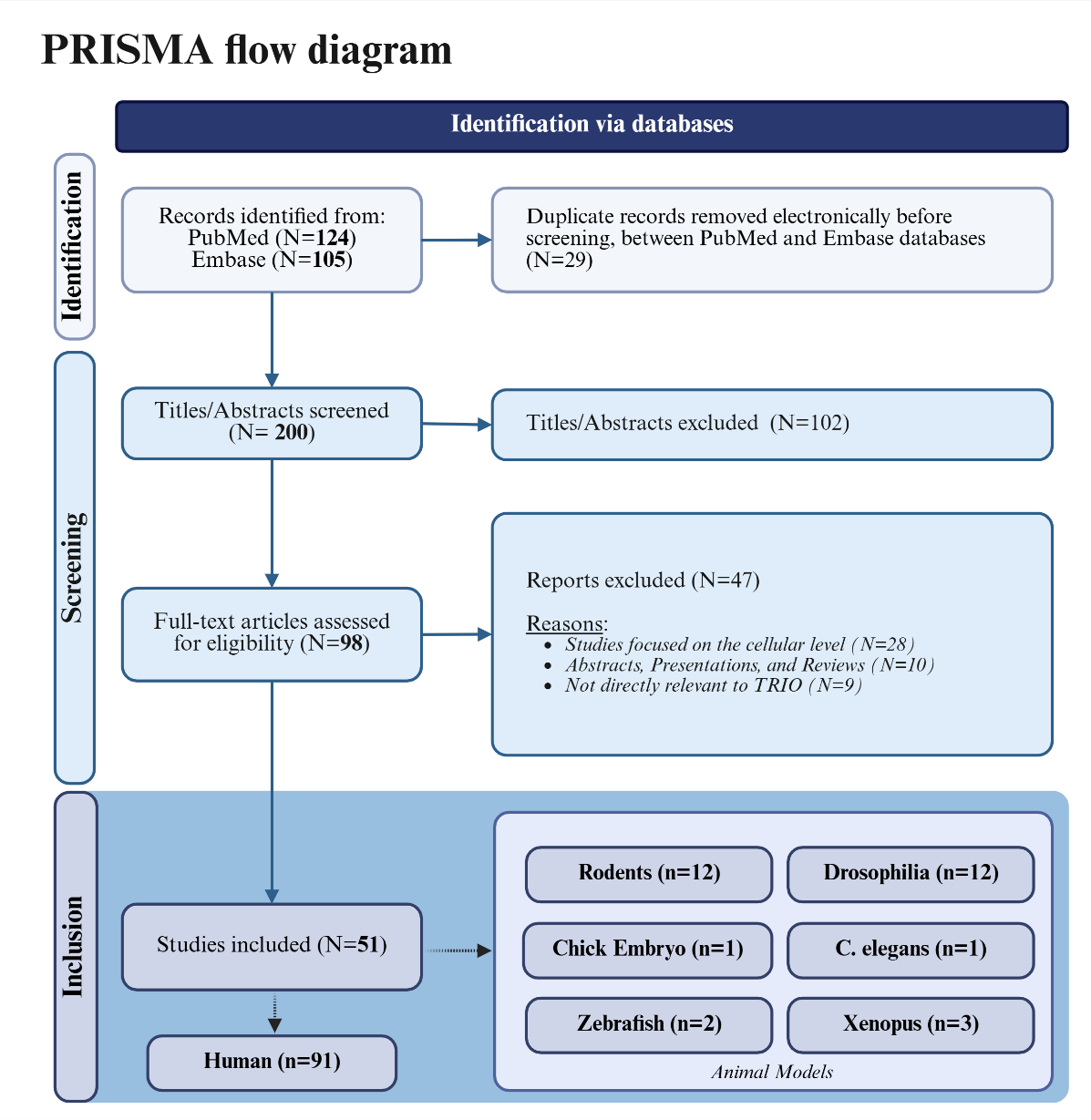
**

***N = number of articles; n = number of patients/animal models**

**Supplemental Table 1. Summary of *TRIO* Variants and Associated Phenotypes**. Phenotypes affecting the cerebral ventricles are bolded and underlined. All data was reported as found in the original manuscripts. *All variants were reported in accordance with Human Genome Variation Society nomenclature where feasible, and are otherwise reported as found in the original manuscripts.

| **Genotype (Variant)*** | **N** | **Sex** | **Variant classification** | **Inheritance** | **Protein Domain** | **Additional Genotype*** | **Neuro**  **Phenotype** | **Reference** |
| --- | --- | --- | --- | --- | --- | --- | --- | --- |
| **c.77C>A**  **p.(Ser26*)** | 1 | M | Pathogenic | Inherited | SEC14 | PIK3CA (VUS) c.11G>C p.(Arg4Pro) | - ASD - DD (severe) - Febrile Seizures - Macrocephaly | Schultz-Rogers *et al.* 2020^1^ |
| **c.649A>T**  **p.(Arg217*)** | 1 | M |  | Inherited |  |  | - ADHD | Ba *et al.* 2016^2^ |
| **c.2302C>T**  **p.(Gln768*)** | 1 | F |  |  |  |  |  | Barbosa *et al.* 2020^3^ |
| **c.2690dupA**  **p.(Gln898Alafs*61)** | 1 | M | Pathogenic |  |  |  | - DD (moderate) - Language Delay - Microcephaly | Schultz-Rogers *et al.* 2020^1^ |
| **c.2926del**  **p.(Gln976Argfs*9)** | 1 | M | Pathogenic | *De novo* | Spectrin |  | - DD (mild) | Gazdagh *et al.* 2023^4^ |
| **c.3211C>G**  **p.(Leu1071Val)** | 2 | M (n=2) | Pathogenic (n=1) | *De novo* (n=2) | Spectrin (n=2) | COL1A1 c.3424-1G>A (n=2) | - DD (severe) (n=2) - Macrocephaly (n=1) | Gazdagh *et al.* 2023 (n=1)^4^  Bonnet *et al.* 2023 (n=1)^5^ |
| **c.3220T>C**  **p.(Cys1074Arg)** | 1 | M |  | *De novo* | Spectrin |  | - Global DD - ID (severe) - Macrocephaly - Seizures | Kloth *et al.* 2021^6^ |
| **c.3224C>T**  **p.(Thr1075Ile)** | 2 | M (n=2) |  | *De novo* (n=1) | Spectrin (n=2) | GMPPA c.1169G>A p.(Arg390Gln) | - DD (severe) (n=1) - Macrocephaly (n=1) - DD (moderate) (n=1) | Kloth *et al.* 2021 (n=1)^6^  Barbosa *et al.* 2020 (n=1)^3^ |
| **c.3229G>C**  **p.(Ala1077Pro)** | 2 | F (n=2) | Pathogenic (n=1) | *De novo* (n=2) | Spectrin (n=2) |  | - DD (severe) (n=1) - ID (severe) (n=1) - Macrocephaly (n=1) - Seizures (n=1) | Gazdagh *et al.* 2023 (n=1)^4^  Bonnet *et al.* 2023 (n=1)^5^ |
| **c.3232C>T**  **p.(Arg1078Trp)** | 13 | F (n=6)  M (n=7) | Pathogenic (n=4) | *De novo* (n=12) | Spectrin (n=13) | 16p13.3 microdeletion (n=1)  14q21.1 microdeletion (n=1)  DYNC1H1 (VUS) (n=2) | - **Hydrocephalus** (n=1) - ADHD (n=2) - Arnold-Chiari Malformation (n=2) - ASD (n=2) - DD (moderate) (n=2) - DD (severe) (n=8) - Global DD (severe) (n=2) - ID (severe) (n=1) - IVH Neonatal (n=1) - Macrocephaly (n=4) - PVL-PVNH (n=1) - Seizures (n=2) - Thin CC (n=1) | Barbosa *et al.* 2020 (n=5)^3^  Bonnet *et al.* 2023 (n=4)^5^ |
| **c.3233G>A**  **p.(Arg1078Gln)** | 2 | F (n=2) |  | *De novo* (n=2) | Spectrin (n=2) |  | - DD (moderate) (n=1) - DD (severe) (n=1) - Seizures (n=2) | Barbosa *et al.* 2020 (n=2)^3^ |
| **c.3239A>T**  **p.(Asn1080Ile)** | 1 | F |  | *De novo* | Spectrin |  | - DD (severe) - Seizures | Barbosa *et al.* 2020^3^ |
| **c.3371T>C**  **p.(Leu1124Ser)** | 2 | F (n=2) |  |  | Spectrin (n=2) |  | - DD (moderate) (n=1) - ID (moderate) (n=1) - Seizures (n=1) | Gazdagh *et al.* 2023 (n=1)^4^  Bonnet *et al.* 2023 (n=1)^5^ |
| **c.3421G>A**  **p.(Val1141Met)** | 2 | F (n=2) |  | *De novo* (n=2) | Spectrin (n=2) | FOXP1 c.1428+1G>C (intron 16) (n=2) | - ASD (n=2) - DD (severe) (n=1) - ID (severe) (n=1) | Gazdagh *et al.* 2023 (n=1)^4^  Bonnet *et al.* 2023 (n=1)^5^ |
| **c.3475G>A**  **p.(Glu1159Lys)** | 2 | F (n=2) | Likely Pathogenic (n=1) | *De novo* (n=2) | Spectrin (n=2) |  | - ADHD (n=2) - ASD (n=2) - DD (moderate) (n=1) - Global DD (n=1) | Gazdagh *et al.* 2023 (n=1)^4^  Bonnet *et al.* 2023 (n=1)^5^ |
| **c.3506delG**  **p.(Gly1169Alafs*11)** | 2 | F (n=1)  M (n=1) |  |  |  |  | - **Widened Left Lateral Ventricle** (n=1) - Delayed development of Cerebral Cortex Cerebellar and Brainstem (n=1) - Shorter Transverse Cerebellar diameter (n=1) | Liu *et al.* 2022 (n=2)^7^ |
| **c.3641C>T  p.(Ala1214Val)** | 6 | F (n=4)  M (n=2) | Likely Pathogenic | Inherited (n=6) | Spectrin (n=6) |  | - ASD (n=1) - DD (n=2) - Dysarthria (n=3) - ID (n=3) - Obsessive behavior (n=1) - Schizophrenia (n=1) - Seizures (n=1) - Spastic ataxia (n=4) | Al Shaikh *et al*. 2018 (n=6)^8^ |
| **c.3727del**  **p.(Ser1244Leufs*23)** | 1 | M | Likely Pathogenic | *De novo* |  |  | - **Ventriculomegaly** - DD (mild) | Gazdagh *et al.* 2023^4^ |
| **c.3752del**  **p.(Asp1251Valfs*11)** | 1 | F |  | *De novo* |  |  | - ASD | Ba *et al.* 2016^2^ |
| **c.3895G>A**  **p.(Glu1299Lys)** | 1 | M |  | *De novo* | GEF1 |  | - DD (moderate) | Barbosa *et al.* 2020^3^ |
| **c.4103A>T**  **p.(Asp1368Val)** | 1 | M |  |  |  |  | - **Ventriculomegaly** - DD - ID (severe) - Speech delay | De Ligt *et al.* 2012^9^ |
| **Chr5(GRCh37):g.14390384A>T p.(Asp1368Val)** | 1 |  |  |  |  |  |  | Gilissen *et al.* 2014^10^ |
| **c.4112A>G**  **p.(His1371Arg)** | 1 | F | Likely Pathogenic | *De novo* | GEF1 |  | - **Dysmorphic lateral ventricle** - DD (moderate) - Dysmorphic Basal Ganglia - Hypoplastic CC - Seizures | Gazdagh *et al.* 2023^4^ |
| **c.4111C>T**  **p.(His1371Tyr)** | 1 | M | Likely Pathogenic | *De novo* | GEF1 |  | - Absent Speech - Epilepsy - ID (severe) - Macrocephaly | Aspromonte *et al.* 2019^11^ |
| **c.4128G>A**  **p.(Trp1376*)** | 1 | M |  | *De novo* |  |  | - ADHD | Ba *et al.* 2016^2^ |
| **c.4231C>T  p.(Arg1411*)** | 2 | F (n=1)  M (n=1) | Likely Pathogenic (n=1)  Pathogenic (n=1) | *De novo* (n=1)  Inherited (maternal) (n=1) | GEF1 (n=1) |  | - ASD (n=1) - DD (moderate) (n=1) - Microcephaly (n=1) | Gazdagh *et al.* 2023 (n=1)^4^  Aspromonte *et al.* 2019 (n=1)^11^ |
| **c.4283G>A**  **p.(Arg1428Gln)** | 6 | F (n=3)  M (n=3) | Pathogenic (n=3) | *De novo* (n=5) | GEF1 (n=6) | SHANK3 (VUS) c.748G>T p.(Gly250Cys) (n=1)  SHANK3 (VUS) c.768G>T p.(Gln256His) (n=1) | - ASD (n=1) - CC Agenesis (n=1) - DD (moderate) (n=1) - DD (severe) (n=1) - Global DD (n=3) - Microcephaly (n=2) | Gazdagh *et al.* 2023 (n=2)^4^  Pengelly *et al.* 2016 (n=1)^12^  Barbosa *et al.* 2020 (n=2)^3^  Bevilacqua et al. 2021 (n=1)^13^ |
| **c.4342G>A**  **p.(Gly1448Arg)** | 2 | M (n=2) | Likely Pathogenic (n=1) | *De novo* (n=1) | GEF1 (n=2) |  | - DD (moderate) (n=1) - ID (severe) (n=1) - Macrocephaly (n=1) | Gazdagh *et al.* 2023 (n=1)^4^  Bonnet *et al.* 2023 (n=1)^5^ |
| **c.4382C>T**  **p.(Pro1461Leu)** | 2 | F (n=2) |  | *De novo* (n=2) | GEF1 (n=2) | FAT4 variants (biallelic) | - ASD (n=2) - DD (mild) (n=1) - DD (moderate) (n=1) | Barbosa *et al.* 2020 (n=2)^3^ |
| **c.4381C>A**  **p.(Pro1461Thr)** | 1 | F |  | *De novo* | GEF1 |  | - Global DD | Barbosa *et al.* 2020^3^ |
| **c.4394A>G**  **p.(Asn1465Ser)** | 2 | F (n=1)  M (n=1) | VUS but functional data provide evidence for pathogenicity  (n=2) |  | GEF1  (n=2) | ZSWIM6 (VUS) c.1165A>C p.(Asn389His) | - DD (mild) (n=1) - Microcephaly (n=1) | Gazdagh *et al.* 2023 (n=2)^4^ |
| **c.4406A>G**  **p.(His1469Arg)** | 1 | M |  | *De novo* | GEF1 |  |  | Barbosa *et al.* 2020^3^ |
| **c.4466delA**  **p.(Gln1489Argfs*12) (n=3)**  **c.4466delA**  **p.(Gln1489Argfs*11) (n=3)** | 6 | F (n=2)  M (n=4) |  | Inherited (n=6) | GEF1 (n=6) | 15q11.2 microdeletion  (n=6)  KCNJ2 (n=2) | - DD (mild) (n=6) - Microcephaly (n=1) | Barbosa *et al.* 2020 (n=3)^3^  Pengelly *et al.* 2016 (n=3)^12^ |
| **c.4589A>G**  **p.(Lys1530Arg)** | 1 | M |  | *De novo* |  |  | - ID - ADHD - Anxiety - Microcephaly | Mainali *et al*. 2023^14^ |
| **c.4860-2A>G**  **p.(Arg1620Serfs*10)** | 1 | F |  | *De novo* |  |  | - DD (mild) - Microcephaly | Barbosa *et al.* 2020^3^ |
| **c.5419del**  **p.(Arg1807Alafs*33)** | 1 | M | Likely Pathogenic |  |  |  | - ASD - DD (mild) - Microcephaly | Gazdagh *et al.* 2023^4^ |
| **c.6092dup**  **p.(Leu2031Phefs*9)** | 1 | M |  | *De novo* |  |  | - DD (severe) - ASD | Barbosa *et al.* 2020^3^ |
| **c.6239T>C**  **p.(Phe2080Ser)** | 1 | F | Pathogenic | *De novo* | GEF2 | POLG variant c.2209G>C p.(Gly737Arg) | - DD (moderate) | Gazdagh *et al.* 2023^4^ |
| **c.6554_6557del**  **p.(Glu2185Glyfs*35)** | 1 | M | Likely Pathogenic | Inherited (maternal) | GEF2 |  | - DD (mild) - Progressive Leukoencephalopathy - Seizures | Gazdagh *et al.* 2023^4^ |
| **c.6995del**  **p.(Ser2332Thrfs*81)** | 1 | F | Pathogenic | *De novo* |  |  | - DD (moderate) - Seizures - Visual problems suggestive of bilateral macular pathway dysfunction with pale optic disks | Gazdagh *et al.* 2023^4^ |
| **c.7050del**  **p.(Val2351Cysfs*62)** | 1 | M |  | *De novo* |  | 5p15.31 microdeletion (VUS) | - DD (moderate) - Seizures | Barbosa *et al.* 2020^3^ |
| **c.7416del**  **p.(Phe2473Serfs*54)** | 1 | M |  |  |  |  | - DD (severe) - Seizures | Barbosa *et al.* 2020^3^ |
| **c.7688C>T**  **p.(Thr2563Met)** | 1 | F |  |  |  |  | - **Ventriculomegaly** - DD - ID (severe) - Speech delay | De Ligt *et al.* 2012^9^ |
| **c.3949-122_4312-240del** | 1 | M | Likely Pathogenic | *De novo* | GEF1 |  | - DD (severe) - Lissencephaly - Seizures | Gazdagh *et al.* 2023^4^ |
| **c.4311+1G>A** | 1 | F |  |  |  |  | - Global DD - Microcephaly | Lionel *et al.* 2018^15^ |
| **c.4716G>A**  **p.(Asn1465Ser)** | 3 | F (n=2)  M (n=1) | Pathogenic (n=3) | Inherited (n=3) | GEF1 (n=3) |  | - NDD (mild) (n=1) - Speech delay (n=2) - ID (mild) (n=1) - Microcephaly (n=2) | Schwartzmann *et al*. 2024 (n=3)^16^ |
| **c.5203+1dup** | 1 | M | Pathogenic | Inherited |  |  | - DD (mild-moderate) | Gazdagh *et al.* 2023^4^ |
| **c.5651A>C** | 1 | M | VUS |  |  | TRPV4 c.880T>G | - DD - Speech delay | Glotov *et al*. 2024^17^ |
| **c.6244-2A>G** | 1 | F | Likely Pathogenic | *De novo* | GEF2 |  | - DD (moderate) - Global Cerebral Atrophy - Secondary Agenesis of Corpus Callosum | Gazdagh *et al.* 2023^4^ |
| **Chr5:14160447-14395478**  **Trio Deleted** | 1 | M |  |  |  |  | - PDD-NOS | Ba *et al.* 2016^2^ |

**Supplemental Table 2. Individual Patient Data: TRIO Gene Variants and Associated Phenotypes** Phenotypes affecting the cerebral ventricles are bolded and underlined. * All variants were reported in accordance with Human Genome Variation Society nomenclature where feasible, and are otherwise reported as found in the original manuscripts.

| **Patient ID** | **Sex** | **Genotype (Variant)*** | **Variant classification** | **Inheritance** | **Protein Domain** | **Additional Genotype*** | **Neuro**  **Phenotype** | **Reference** |
| --- | --- | --- | --- | --- | --- | --- | --- | --- |
| 1 | M | c.77C>A  p.(Ser26*) | Pathogenic | Inherited | SEC14 | PIK3CA (VUS) c.11G>C p.(Arg4Pro) | - DD (severe) - ASD - Stereotypies - Macrocephaly - Febrile Seizures - Dysmorphism | Schultz-Rogers *et al.* 2020^1^ |
| 2 | M | c.649A>T  p.(Arg217*) |  | Inherited |  |  | - ADHD | Ba *et al.* 2016^2^ |
| 3 | F | c.2302C>T  p.(Gln768*) |  | - |  |  |  | Barbosa *et al.* 2020^3^ |
| 4 | M | c.2690dupA  p.(Gln898Alafs*61) | Pathogenic |  |  |  | - DD (moderate) - Language Delay - Microcephaly - Cutis Aplasia - Dysmorphism | Schultz-Rogers *et al.* 2020^1^ |
| 5 | M | c.2926del  p.(Gln976Argfs*9) | Pathogenic | *De novo* | Spectrin |  | - DD (mild) | Gazdagh *et al.* 2023^4^ |
| 6 | M | c.3211C>G  p.(Leu1071Val) | Pathogenic | *De novo* | Spectrin | COL1A1  c.3424-1G>A | - DD (severe) | Gazdagh *et al.* 2023^4^ |
| 7 | M | c.3211C>G  p.(Leu1071Val) |  | *De novo* | Spectrin | COL1A1 variant | - DD (severe) - Macrocephaly | Bonnet *et al.* 2023^5^ |
| 8 | M | c.3220T>C  p.(Cys1074Arg) |  | *De novo* | Spectrin |  | - Global DD - ID (severe) - Seizures - Macrocephaly | Kloth *et al.* 2021^6^ |
| 9 | M | c.3224C>T  p.(Thr1075Ile) |  | - | Spectrin |  | - DD (moderate) | Barbosa *et al.* 2020^3^ |
| 10 | M | c.3224C>T  p.(Thr1075Ile) |  | *De novo* | Spectrin | GMPPA c.1169G>A p.(Arg390Gln) | - DD (severe) - Dystrophy - Muscular Hypotonia and Stereotypic Limb Movements - Secondary Relative Macrocephaly | Kloth *et al.* 2021^6^ |
| 11 | F | c.3229G>C  p.(Ala1077Pro) | Pathogenic | *De novo* | Spectrin |  | - DD (severe) - Seizures | Gazdagh *et al.* 2023^4^ |
| 12 | F | c.3229G>C  p.(Ala1077Pro) |  | *De novo* | Spectrin |  | - ID (severe) - Macrocephaly | Bonnet *et al.* 2023^5^ |
| 13 | M | c.3232C>T  p.(Arg1078Trp) |  | *De novo* | Spectrin | 14q21.1 microdeletion | - DD (severe) | Barbosa *et al.* 2020^3^ |
| 14 | M | c.3232C>T  p.(Arg1078Trp) |  | *De novo* | Spectrin |  | - DD (severe) | Barbosa *et al.* 2020^3^ |
| 15 | M | c.3232C>T  p.(Arg1078Trp) |  | *De novo* | Spectrin |  | - DD (severe) | Barbosa *et al.* 2020^3^ |
| 16 | M | c.3232C>T  p.(Arg1078Trp) |  | *De novo* | Spectrin |  | - DD (severe) | Barbosa *et al.* 2020^3^ |
| 17 | M | c.3232C>G  p.(Arg1078Gly) |  | *De novo* | Spectrin |  | - DD (severe) | Barbosa *et al.* 2020^3^ |
| 18 | F | c.3233G>A  p.(Arg1078Gln) |  | *De novo* | Spectrin |  | - DD (moderate) - Seizures | Barbosa *et al.* 2020^3^ |
| 19 | F | c.3233G>A  p.(Arg1078Gln) |  | *De novo* | Spectrin |  | - DD (severe) - Seizures | Barbosa *et al.* 2020^3^ |
| 20 | M | c.3232C>T  p.(Arg1078Trp) | Pathogenic | *De novo* | Spectrin |  | - DD (severe) - Seizures - IVH Neonatal - **Hydrocephalus** | Gazdagh *et al.* 2023^4^ |
| 21 | F | c.3232C>T  p.(Arg1078Trp) | Pathogenic | *De novo* | Spectrin |  | - DD (severe) - Arnold-Chiari Malformation - Growth Retardation - Dysmorphism | Gazdagh *et al.* 2023^4^ |
| 22 | F | c.3232C>T  p.(Arg1078Trp) | Pathogenic | *De novo* | Spectrin | 16p13.3 microdeletion  DYNC1H1 (VUS) | - DD (moderate) - ASD - ADHD - Tremor | Gazdagh *et al.* 2023^4^ |
| 23 | F | c.3232C>T  p.(Arg1078Trp) | Pathogenic | *De novo* | Spectrin |  | - DD (severe) - Thin CC - Delayed Myeling Macrocephaly - Seizures | Gazdagh *et al.* 2023^4^ |
| 24 | F | c.3232C>T  p.(Arg1078Trp) |  | *De novo* | Spectrin |  | - ID (severe) - Macrocephaly | Bonnet *et al.* 2023^5^ |
| 25 | F | c.3232C>T  p.(Arg1078Trp) |  | *De novo* | Spectrin | DYNC1H1 (VUS) | - ASD - ADHD - Moderate Global DD - PVL-PVNH | Bonnet *et al.* 2023^5^ |
| 26 | F | c.3232C>T  p.(Arg1078Trp) |  |  | Spectrin |  | - Global DD (severe) - Moderate Hypotonia - Arnold-Chiari Malformation - Macrocephaly | Bonnet *et al.* 2023^5^ |
| 27 | M | c.3232C>T  p.(Arg1078Trp) |  | *De novo* | Spectrin |  | - Global DD (severe) - Macrocephaly | Bonnet *et al.* 2023^5^ |
| 28 | F | c.3239A>T  p.(Asn1080Ile) |  | *De novo* | Spectrin |  | - DD (severe) - Seizures | Barbosa *et al.* 2020^3^ |
| 29 | F | c.3371T>C  p.(Leu1124Ser) |  |  | Spectrin |  | - DD (moderate) - Myoclonus Dystonia | Gazdagh *et al.* 2023^4^ |
| 30 | F | c.3371T>C  p.(Leu1124Ser) |  |  | Spectrin |  | - ID (moderate) - Seizures - Dystonia | Bonnet *et al.* 2023^5^ |
| 31 | F | c.3421G>A  p.(Val1141Met) |  | *De novo* | Spectrin | FOXP1 c.1428+1G>C (intron 16) | - DD (severe) - Hyperactivity ASD | Gazdagh *et al.* 2023^4^ |
| 32 | F | c.3421G>A  p.(Val1141Met) |  | *De novo* | Spectrin | FOXP1 c.1428+1G>C (intron 16) | - ID (severe) - ASD | Bonnet *et al.* 2023^5^ |
| 33 | F | c.3475G>A  p.(Glu1159Lys) | Likely Pathogenic | *De novo* | Spectrin |  | - DD (moderate) - ASD - ADHD | Gazdagh *et al.* 2023^4^ |
| 34 | F | c.3475G>A  p.(Glu1159Lys) |  | *De novo* | Spectrin |  | - Global DD - ADHD - ASD - Hypotonia | Bonnet *et al.* 2023^5^ |
| 35 | M | c.3506delG  p.(Gly1169Alafs*11) |  |  |  |  | - Shorter Transverse Cerebellar diameter - Delayed development of Cerebral Cortex Cerebellar and Brainstem | Liu *et al.* 2022^7^ |
| 36 | F | c.3506delG  p.(Gly1169Alafs*11) |  |  |  |  | - **Widened Left Lateral Ventricle** | Liu *et al.* 2022^7^ |
| 37 | F | c.3641C>T p.(Ala1214Val) | Likely Pathogenic | *Inherited* | Spectrin |  | - Seizures - Cognitive decline - Spastic ataxia - Dysarthria | Al Shaikh *et al*. 2018^8^ |
| 38 | F | c.3641C>T p.(Ala1214Val) | Likely Pathogenic | *Inherited* | Spectrin |  | - ID - Schizophrenia - ASD - Ataxia - Dysarthria | Al Shaikh *et al*. 2018^8^ |
| 39 | F | c.3641C>T p.(Ala1214Val) | Likely Pathogenic | *Inherited* | Spectrin |  | - Cognitive impairment - Spastic ataxia - Obsessive behavior | Al Shaikh *et al*. 2018^8^ |
| 40 | M | c.3641C>T  p.(Ala1214Val) | Likely Pathogenic | *Inherited* | Spectrin |  | - Mild ID - Speech disorder - Ataxia - Dysarthria | Al Shaikh *et al*. 2018^8^ |
| 41 | F | c.3641C>T p.(Ala1214Val) |  | *Inherited* | Spectrin |  |  | Al Shaikh *et al*. 2018^8^ |
| 42 | M | c.3641C>T p.(Ala1214Val) |  | *Inherited* | Spectrin |  |  | Al Shaikh *et al*. 2018^8^ |
| 43 | M | c.3727del  p.(Ser1244Leufs*23) | Likely Pathogenic | *De novo* |  |  | - DD (mild) - Dysmorphism - **Ventriculomegaly** - Myopia | Gazdagh *et al.* 2023^4^ |
| 44 | F | c.3752del  p.(Asp1251Valfs*11) |  | *De novo* |  |  | - ASD | Ba *et al.* 2016^2^ |
| 45 | M | c.3895G>A  p.(Glu1299Lys) |  | *De novo* | GEF1 |  | - DD (moderate) | Barbosa *et al.* 2020^3^ |
| 46 |  | Chr5(GRCh37):g.14390384A>T p.(Asp1368Val) |  |  |  |  |  | Gilissen *et al.* 2014^10^ |
| 47 | M | c.4103A>T p.(Asp1368Val) |  |  |  |  | - ID (severe) - DD - Speech delay - **Ventriculomegaly** | De Ligt *et al.* 2012^9^ |
| 48 | M | c.4111C>T  p.(His1371Tyr) | Likely Pathogenic | *De novo* | GEF1 |  | - ID (severe) - Epilepsy - Absent Speech - Macrocephaly | Aspromonte *et al.* 2019^11^ |
| 49 | F | c.4112A>G  p.(His1371Arg) | Likely Pathogenic | *De novo* | GEF1 |  | - DD (moderate) - Seizures - Hypoplastic CC - **Dysmorphic lateral ventricle** - Dysmorphic Basal Ganglia | Gazdagh *et al.* 2023^4^ |
| 50 | M | c.4128G>A  p.(Trp1376*) |  | *De novo* |  |  | - ADHD | Ba *et al.* 2016^2^ |
| 51 | M | c.4231C>T  p.(Arg1411*) | Pathogenic | Inherited (maternal) |  |  | - Microcephaly | Aspromonte *et al.* 2019^11^ |
| 52 | F | c.4231C>T  p.(Arg1411*) | Likely Pathogenic | *De novo* | GEF1 | arr[GRCh37] 6q13q14.1 (72983442_77064061)x3  arr[GRCh37] 18p11.32p11.21 (136226_15054986)x1  arr[GRCh37] 19p13.3 (685215_1334735)x3 | - DD (moderate) - ASD | Gazdagh *et al.* 2023^4^ |
| 53 | F | c.4283G>A  p.(Arg1428Gln) |  | *De novo* | GEF1 |  |  | Barbosa *et al.* 2020^3^ |
| 54 | F | c.4283G>A  p.(Arg1428Gln) |  | *De novo* | GEF1 |  | - DD (severe) | Barbosa *et al.* 2020^3^ |
| 55 | M | c.4283G>A  p.(Arg1428Gln) | Pathogenic | *De novo* | GEF1 | SHANK3 (VUS)  c.748G>T  p.(Gly250Cys)  SHANK3 (VUS)  c.768G>T  p.(Gln256His) | - Global DD - Microcephaly - ASD | Gazdagh *et al.* 2023^4^ |
| 56 | M | c.4283G>A  p.(Arg1428Gln) | Pathogenic | *De novo* | GEF1 |  | - DD (moderate) | Gazdagh *et al.* 2023^4^ |
| 57 | F | c.4283G>A  p.(Arg1428Gln) |  | *De novo* | GEF1 |  | - Global DD - Corpus Callosum Agenesis | Pengelly *et al.* 2016^12^ |
| 58 | M | c.4283G>A p.(Arg1428Gln) | Pathogenic |  | GEF1 |  | - Global DD - Microcephaly | Bevilacqua *et al.* 2021^13^ |
| 59 | M | c.4342G>A  p.(Gly1448Arg) | Likely Pathogenic | *De novo* | GEF1 |  | - DD (moderate) - Macrocephaly | Gazdagh *et al.* 2023^4^ |
| 60 | M | c.4342G>A  p.(Gly1448Arg) |  |  | GEF1 |  | - ID (severe) | Bonnet *et al.* 2023^5^ |
| 61 | F | c.4381C>A  p.(Pro1461Thr) |  | *De novo* | GEF1 |  | - Global DD | Barbosa *et al.* 2020^3^ |
| 62 | F | c.4382C>T  p.(Pro1461Leu) |  | *De novo* | GEF1 |  | - DD (mild) - ASD | Barbosa *et al.* 2020^3^ |
| 63 | F | c.4382C>T  p.(Pro1461Leu) |  | *De novo* | GEF1 | FAT4 variants (biallelic) | - DD (moderate) - ASD | Barbosa *et al.* 2020^3^ |
| 64 | F | c.4394A>G  p.(Asn1465Ser) | VUS but functional data provide evidence for pathogenicity |  | GEF1 | ZSWIM6 (VUS) c.1165A>C p.(Asn389His) | - Microcephaly | Gazdagh *et al.* 2023^4^ |
| 65 | M | c.4394A>G  p.(Asn1465Ser) | VUS but functional data provide evidence for pathogenicity |  | GEF1 |  | - DD (mild) | Gazdagh *et al.* 2023^4^ |
| 66 | M | c.4406A>G  p.(His1469Arg) |  | *De novo* | GEF1 |  |  | Barbosa *et al.* 2020^3^ |
| 67 | F | c.4466delA  p.(Gln1489Argfs*12) |  | Inherited | GEF1 | 15q11.2 microdeletion | - DD (mild) | Barbosa *et al.* 2020^3^ |
| 68 | M | c.4466delA  p.(Gln1489Argfs*12) |  | Inherited | GEF1 | 15q11.2 microdeletion | - DD (mild) | Barbosa *et al.* 2020^3^ |
| 69 | M | c.4466delA  p.(Gln1489Argfs*12) |  | Inherited | GEF1 | KCNJ2 variant  15q11.2 microdeletion | - DD (mild) | Barbosa *et al.* 2020^3^ |
| 70 | F | c.4466delA  p.(Gln1489Argfs*11) |  | Inherited | GEF1 | 15q11.2 microdeletion | - DD (mild) | Pengelly *et al.* 2016^12^ |
| 71 | M | c.4466delA  p.(Gln1489Argfs*11) |  | Inherited | GEF1 | 15q11.2 microdeletion | - DD (mild) - Microcephaly | Pengelly *et al.* 2016^12^ |
| 72 | M | c.4466delA  p.(Gln1489Argfs*11) |  | Inherited | GEF1 | KCNJ2  p.(Thr75Met)  15q11.2 microdeletion | - DD (mild) | Pengelly *et al.* 2016^12^ |
| 73 | M | c.4589A>G  p.(Lys1530Arg) |  | *De novo* |  |  | - ID - ADHD - Anxiety - Microcephaly | Mainali *et al.* 2023^14^ |
| 74 | F | c.4860-2A>G  p.(Arg1620Serfs*10) |  | *De novo* |  |  | - DD (mild) - Microcephaly | Barbosa *et al.* 2020^3^ |
| 75 | M | c.5419del  p.(Arg1807Alafs*33) | Likely Pathogenic |  |  |  | - DD (mild) - Microcephaly - ASD | Gazdagh *et al.* 2023^4^ |
| 76 | M | c.6092dup  p.(Leu2031Phefs*9) |  | *De novo* |  |  | - DD (severe) - ASD | Barbosa *et al.* 2020^3^ |
| 77 | F | c.6239T>C  p.(Phe2080Ser) | Pathogenic | *De novo* | GEF2 | POLG c.2209G>C p.(Gly737Arg) | - DD (moderate) | Gazdagh *et al.* 2023^4^ |
| 78 | M | c.6554_6557del  p.(Glu2185Glyfs*35) | Likely Pathogenic | Inherited (maternal) | GEF2 |  | - DD (mild) - Seizures - Progressive Leukoencephalopathy | Gazdagh *et al.* 2023^4^ |
| 79 | F | c.6995del  p.(Ser2332Thrfs*81) | Pathogenic | *De novo* |  |  | - DD (moderate) - Seizures - Visual Problems suggestive of Bilateral Macular Pathway Dysfunction with Pale Optic Disks | Gazdagh *et al.* 2023^4^ |
| 80 | M | c.7050del  p.(Val2351Cysfs*62) |  | *De novo* |  | 5p15.31 microdeletion (VUS) | - DD (moderate) - Seizures | Barbosa *et al.* 2020^3^ |
| 81 | M | c.7416del  p.(Phe2473Serfs*54) |  |  |  |  | - DD (severe) - Seizures | Barbosa *et al.* 2020^3^ |
| 82 | F | c.7688C>T p.(Thr2563Met) |  |  |  |  | - ID (severe) - DD - Speech delay - **Ventriculomegaly** | De Ligt *et al.* 2012^9^ |
| 83 | M | c.3949-122_4312-240del | Likely Pathogenic | *De novo* | GEF1 |  | - DD (severe) - Seizures - Lissencephaly | Gazdagh *et al.* 2023^4^ |
| 84 | M | c.5203+1dup | Pathogenic | Inherited |  |  | - DD (mild-moderate) | Gazdagh *et al.* 2023^4^ |
| 85 | F | c.6244-2A>G | Likely Pathogenic | *De novo* | GEF2 |  | - DD (moderate) - Global Cerebral Atrophy - Secondary Agenesis of Corpus Callosum | Gazdagh *et al.* 2023^4^ |
| 86 | M | Chr5:14160447-14395478  Trio Deleted |  |  |  |  |  | Ba *et al.* 2016^2^ |
| 87 | F | c.4311+1G>A |  |  |  |  | - Microcephaly - Global DD | Lionel *et al.* 2018^15^ |
| 88 | F | c.4716G>A  p.(Asn1465Ser) | Pathogenic | Inherited | GEF1 |  | - Mild NDD, dyslexia - Microcephaly | Schwartzmann *et al.* 2024^16^ |
| 89 | M | c.4716G>A  p.(Asn1465Ser) | Pathogenic | Inherited | GEF1 |  | - Mild ID - Microcephaly | Schwartzmann *et al.* 2024^16^ |
| 90 | F | c.4716G>A  p.(Asn1465Ser) | Pathogenic | Inherited | GEF1 |  | - Speech delay - DD - Microcephaly | Schwartzmann *et al.* 2024^16^ |
| 91 | M | c.5651A>C | VUS |  |  | TRPV4 c.880T>G | - DD - Speech delay - Inability to sit/walk - Joint contractures | Glotov *et al*. 2024^17^ |

**Supplemental Table 3. TRIO Animal Models and Phenotypes.** Phenotypes affecting the cerebral ventricles are bolded and underlined. All data was reported as found in the original manuscripts.

| **Species** | **Genetic Model (Manipulation)** | **Phenotype** | **Reference** |
| --- | --- | --- | --- |
| **Mouse** | **Trio** knockout (Trio -/-) | - Embryonic lethal ~E15.5–birth - Abnormal skeletal muscle development (spherical secondary myofibers) - Disorganized hippocampus/olfactory bulb. - Misrouted thalamocortical axons (TCA), defective growth cone collapse, mispositioned corridor cells, reduced RhoA activation - Impaired GABAergic interneuron (IN) migration | O’Brien et al. 2000^18^  Backer *et al*. 2007^19^  Eid *et al.* 2025^20^ |
|  | **Trio** heterozygous (Trio +/-) | - Viable with neurodevelopmental deficits - Smaller brain - Reduced dendritic arborisation - Behavioral deficits (social, anxiety, motor) | Katrancha et al. 2019^21^ |
|  | **Trio** cKO (Nestin-Cre; Trio ^flox/flox^) | - Most pups die at birth - Survivors have cerebellar ataxia - No internal granule layer - Reduced brain size. | Peng et al. 2010^22^ |
|  | **Trio** cKO (Emx1-Cre; Trio^flox/flox^) | - Survives to adulthood with reduced brain size - Abnormal hippocampal organization - Impaired spatial learning | Zong et al. 2015^23^ |
|  | **Trio** cKO (Neurod6-Cre; Trio^flox/flox^) | - Viable with reduced brain volume - Decreased dendritic complexity in cortical pyramidal neurons - Cognitive and motor deficits | Katrancha et al. 2019^21^ |
|  | **Trio** cKO (Wnt1-Cre; Trio^flox/flox^) | - Craniofacial defects (mandibular retrusion, widened sutures) - Impaired NCC migration and differentiation | Guo *et al.* 2021^24^ |
|  | **Trio** variant KI (Trio+/K1431M, +/K1918X, or +/M2145T) | - Viable - Variant-specific mild neurodevelopmental and behavioral phenotypes - Subtle reductions in brain/synapse structure | Ishchenko et al. 2024^25^ |
|  | **Trio cKO (Dlx5/6Cre;Triolox/lox)** | - Spontaneous seizures - ASD-like behavior - Reduced PV+, CR+, VIP+ interneurons - Cortical inhibition deficit | Eid et al. 2025^20^ |
|  | GEF1-only or GEF2-only rescue | - Partial rescue of migration/branching - Both GEF1 (Rac1) and GEF2 (RhoA) required for full rescue | Eid *et al.* 2025^20^ |
|  | Trio/Tiam1 inhibition | - Neurite retraction and apoptosis of ESC-derived motor neurons via ERK5/AKT deactivation and caspase-3 activation | Stankiewicz *et al*. 2020^26^ |
|  | **Rac1** forebrain progenitor KO (Emx1- or Foxg1-Cre; Rac1^flox/flox^) | - Microcephaly - Agenesis of corpus callosum - Disorganized lamination - **Enlarged lateral ventricles** | Chen et al. 2009^27^ |
| **Rat** | **Trio isoforms (Trio 8, 9S, 9L, and Trio/duet)** | - Differential expression of multiple Trio isoforms in brain regions (cortex, cerebellum), with isoform-specific and developmentally regulated expression patterns. | McPherson *et al*. 2005^28^ |
| **Chick embryo** | **Tripα overexpression (inhibits Trio)** | - Loss of apical constriction during lens pit formation - Defective epithelial invagination - Rescued by RhoA activation | Plageman *et al*. 2011  22031541 |
| **Zebrafish** | **trio** knockdown (morpholino antisense) | - Impaired NCC migration - Craniofacial skeletal deficiencies (mandibular retrusion) - Partially rescued by myh9 or caRac1/caCdc42 | Guo *et al*. 2021^24^ |
|  | **Endothelial cell trio loss (vascular model; Tgfli1 CRISPR)** | - Defective arterial remodeling - Impaired Vegf-induced endothelial expansion - Impaired Rac1/RhoG activation | Klems et al. 2020^29^ |
| **Xenopus** | **trio (cranial neural crest morpholino)** | - Disrupted NC cell migration - Excessive blebbing - Defective craniofacial cartilage - Rescued by Trio GEF2 or Dishevelled | Kratzer *et al.* 2020^30^ |
|  | trio mutant mRNA overexpression (embryo model) | - Spectrin-domain mutant → macrocephaly - GEF1 mutant → microcephaly - Recapitulation of human variant-specific brain defects | Barbosa et al. 2020^3^ |
|  | trio expression study | - Trio mRNA maternally expressed in NCC, somites, cranial nerves - Predicts role in brain and NCC development | Kratzer *et al*. 2019^31^ |
| **Drosophila** | **trio** null mutant | - Embryonic/larval lethal - Axon guidance defects (stalling/misdirection) - Mushroom body malformation | Awasaki et al. 2000^32^  Shivalkar & Giniger, 2012^33^  Newsome *et al*. 2000^34^ |
|  | trio loss-of-function (ISNb/SNa misrouting) | - Motor nerves fail to reach targets - Axon stalling - Mushroom body defects | Brown *et al.* 2017^35^  Kannan *et al.* 2017^36^  Bateman *et al*. 2000^37^ |
|  | trio RNAi knockdown (adult-onset) | - Premature synaptic bouton fragmentation, reduced bouton size, accelerated decline in motor ability | Banerjee *et al.* 2024^38^ |
|  | trio overexpression (Trio-OE) | - Preserved synaptic structure with aging, postponed motor decline, maintained high-frequency neurotransmission, Rac GEF1-dependent | Banerjee *et al.* 2024^38^  Bateman *et al.* 2000^37^ |
|  | GEF1 mutant trio (mutGEF1) | - Failed to prevent bouton fragmentation or preserve motor function, confirming requirement of Rac GEF1 domain | Banerjee *et al.* 2024^38^ |
|  | Human trio transgene | - Functionally rescued synaptic structure in knockdown model; preserved motor function; effect conserved across species | Banerjee *et al.* 2024^38^ |
|  | trio hypomorphic mutant | - Reduced survival to pupal stage, moderately lethal - Mild CNS axon pathfinding defects | Dean *et al.* 2013^39^  Liebl *et* *al*. 2000^40^ |
|  | trio + Abl mutants | - Severe CNS disruption, axon guidance defects, reduced viability | Liebl *et al.* 2000^40^  Forsthoefel *et al.* 2005^41^ |
|  | Rescue with trio cDNA | - Restored normal axon scaffold in double mutant embryos | Liebl *et al*. 2000^40^ |
|  | GEF1 overexpression | - Severe axon misrouting, lamina bypass, axons stall or grow over brain | Newsome *et al*. 2000^34^ |
|  | GEF2 overexpression | - No phenotype | Newsome *et al*. 2000^34^ |
|  | trio + dock / Pak loss-of-function | - Enhanced guidance defects, medulla bypass phenotype | Newsome *et al*. 2000^34^ |
| **C. elegans** | **unc-73** null mutant (Trio homolog) | - Severe axon guidance and cell migration defects - Uncoordinated movement phenotype ("Unc") - Axons fail to reach targets - Impaired excretory canal outgrowth; reduced posterior extension; UNC-73 acts both cell-autonomously (via RhoGEF) and non-autonomously (via RacGEF) - Movement defects due to Rac GEF pathway failure - Guidance errors in VD/DD motor neurons | Watari-Goshima et al. 2007  Norris et al. 2014^42^  Steven *et al.* 2005^43^  Alexander *et al.*  2009^44^  Vanderzalm *et al.* 2009^45^  Kishore *et al.* 2002^46^  Marcus-Gueret *et al.* 2012^47^  Williams *et al.* 2007^48^ |

**References**

1. Schultz-Rogers, L., Muthusamy, K., Pinto E Vairo, F., Klee, E. W. & Lanpher, B. Novel loss-of-function variants in TRIO are associated with neurodevelopmental disorder: case report. *BMC Med. Genet.* **21**, 219 (2020).

2. Ba, W. *et al.* TRIO loss of function is associated with mild intellectual disability and affects dendritic branching and synapse function. *Hum. Mol. Genet.* **25**, 892–902 (2016).

3. Barbosa, S. *et al.* Opposite modulation of RAC1 by mutations in TRIO is associated with distinct, domain-specific neurodevelopmental disorders. *Am. J. Hum. Genet.* **106**, 338–355 (2020).

4. Gazdagh, G. *et al.* Extending the phenotypes associated with TRIO gene variants in a cohort of 25 patients and review of the literature. *Am. J. Med. Genet. A* **191**, 1722–1740 (2023).

5. Bonnet, M. *et al.* Pathogenic TRIO variants associated with neurodevelopmental disorders perturb the molecular regulation of TRIO and axon pathfinding in vivo. *Mol. Psychiatry* **28**, 1527–1544 (2023).

6. Kloth, K. *et al.* More evidence on TRIO missense mutations in the spectrin repeat domain causing severe developmental delay and recognizable facial dysmorphism with macrocephaly. *Neurogenetics* **22**, 221–224 (2021).

7. Liu, Y., Liang, Z., Cai, W., Shao, Q. & Pan, Q. Case report: Phenotype expansion and analysis of TRIO and CNKSR2 variations. *Front. Neurol.* **13**, 948877 (2022).

8. Hanna Al Shaikh, R. *et al.* TRIO gene segregation in a family with cerebellar ataxia. *Neurol. Neurochir. Pol.* **52**, 743–749 (2018).

9. de Ligt, J. *et al.* Diagnostic exome sequencing in persons with severe intellectual disability. *N. Engl. J. Med.* **367**, 1921–1929 (2012).

10. Gilissen, C. *et al.* Genome sequencing identifies major causes of severe intellectual disability. *Nature* **511**, 344–347 (2014).

11. Aspromonte, M. C. *et al.* Characterization of intellectual disability and autism comorbidity through gene panel sequencing. *Hum. Mutat.* **40**, 1346–1363 (2019).

12. Pengelly, R. J. *et al.* Mutations specific to the Rac-GEF domain of TRIO cause intellectual disability and microcephaly. *J. Med. Genet.* **53**, 735–742 (2016).

13. Bevilacqua, F. *et al.* TRIO-related intellectual disability with microcephaly: a case report of a patient with novel clinical findings. *Clin. Dysmorphol.* **30**, 22–26 (2021).

14. Mainali, A. *et al.* Diagnostic yield of clinical exome sequencing in adulthood in medical genetics clinics. *Am. J. Med. Genet. A* **191**, 510–517 (2023).

15. Lionel, A. C. *et al.* Improved diagnostic yield compared with targeted gene sequencing panels suggests a role for whole-genome sequencing as a first-tier genetic test. *Genet. Med.* **20**, 435–443 (2018).

16. Schwartzmann, S. *et al.* RNA analysis and computer-aided facial phenotyping help to classify a novel TRIO splice site variant. *Am. J. Med. Genet. A* **194**, e63599 (2024).

17. Glotov, O. S. *et al.* The benefits of whole-exome sequencing in the differential diagnosis of hypophosphatasia. *Int. J. Mol. Sci.* **25**, (2024).

18. O’Brien, S. P. *et al.* Skeletal muscle deformity and neuronal disorder in Trio exchange factor-deficient mouse embryos. *Proc. Natl. Acad. Sci. U. S. A.* **97**, 12074–12078 (2000).

19. Backer, S. *et al.* Trio GEF mediates RhoA activation downstream of Slit2 and coordinates telencephalic wiring. *Development* **145**, dev153692 (2018).

20. Eid, L. *et al.* Both GEF domains of the autism and developmental epileptic encephalopathy-associated Trio protein are required for proper tangential migration of GABAergic interneurons. *Mol. Psychiatry* **30**, 1338–1358 (2025).

21. Katrancha, S. M. *et al.* Trio haploinsufficiency causes neurodevelopmental disease-associated deficits. *Cell Rep.* **26**, 2805-2817.e9 (2019).

22. Peng, Y.-J. *et al.* Trio is a key guanine nucleotide exchange factor coordinating regulation of the migration and morphogenesis of granule cells in the developing cerebellum. *J. Biol. Chem.* **285**, 24834–24844 (2010).

23. Zong, W. *et al.* Trio gene is required for mouse learning ability. *Brain Res.* **1608**, 82–90 (2015).

24. Guo, S. *et al.* Trio cooperates with Myh9 to regulate neural crest-derived craniofacial development. *Theranostics* **11**, 4316–4334 (2021).

25. Ishchenko, Y. *et al.* Heterozygosity for neurodevelopmental disorder-associated TRIO variants yields distinct deficits in behavior, neuronal development, and synaptic transmission in mice. *bioRxivorg* (2024) doi:10.1101/2024.01.05.574442.

26. Stankiewicz, T. R., Pena, C., Bouchard, R. J. & Linseman, D. A. Dysregulation of Rac or Rho elicits death of motor neurons and activation of these GTPases is altered in the G93A mutant hSOD1 mouse model of amyotrophic lateral sclerosis. *Neurobiol. Dis.* **136**, 104743 (2020).

27. Chen, L., Melendez, J., Campbell, K., Kuan, C.-Y. & Zheng, Y. Rac1 deficiency in the forebrain results in neural progenitor reduction and microcephaly. *Dev. Biol.* **325**, 162–170 (2009).

28. McPherson, C. E., Eipper, B. A. & Mains, R. E. Multiple novel isoforms of Trio are expressed in the developing rat brain. *Gene* **347**, 125–135 (2005).

29. Klems, A. *et al.* The GEF Trio controls endothelial cell size and arterial remodeling downstream of Vegf signaling in both zebrafish and cell models. *Nat. Commun.* **11**, 5319 (2020).

30. Kratzer, M.-C. *et al.* The Rho guanine nucleotide exchange factor Trio is required for neural crest cell migration and interacts with Dishevelled. *Development* **147**, dev.186338 (2020).

31. Kratzer, M.-C., England, L., Apel, D., Hassel, M. & Borchers, A. Evolution of the Rho guanine nucleotide exchange factors Kalirin and Trio and their gene expression in Xenopus development. *Gene Expr. Patterns* **32**, 18–27 (2019).

32. Awasaki, T. *et al.* The Drosophila trio plays an essential role in patterning of axons by regulating their directional extension. *Neuron* **26**, 119–131 (2000).

33. Shivalkar, M. & Giniger, E. Control of dendritic morphogenesis by Trio in Drosophila melanogaster. *PLoS One* **7**, e33737 (2012).

34. Newsome, T. P. *et al.* Trio combines with dock to regulate Pak activity during photoreceptor axon pathfinding in Drosophila. *Cell* **101**, 283–294 (2000).

35. Brown, H. E. *et al.* The function of Drosophila larval class IV dendritic arborization sensory neurons in the larval-pupal transition is separable from their function in mechanical nociception responses. *PLoS One* **12**, e0184950 (2017).

36. Kannan, R. *et al.* The Abl pathway bifurcates to balance Enabled and Rac signaling in axon patterning in Drosophila. *Development* **144**, 487–498 (2017).

37. Bateman, J., Shu, H. & Van Vactor, D. The guanine nucleotide exchange factor trio mediates axonal development in the Drosophila embryo. *Neuron* **26**, 93–106 (2000).

38. Banerjee, S. *et al.* Trio preserves motor synapses and prolongs motor ability during aging. *Cell Rep.* **43**, 114256 (2024).

39. Dean, K. E. *et al.* An allele of sequoia dominantly enhances a trio mutant phenotype to influence Drosophila larval behavior. *PLoS One* **8**, e84149 (2013).

40. Liebl, E. C. *et al.* Dosage-sensitive, reciprocal genetic interactions between the Abl tyrosine kinase and the putative GEF trio reveal trio’s role in axon pathfinding. *Neuron* **26**, 107–118 (2000).

41. Forsthoefel, D. J., Liebl, E. C., Kolodziej, P. A. & Seeger, M. A. The Abelson tyrosine kinase, the Trio GEF and Enabled interact with the Netrin receptor Frazzled in Drosophila. *Development* **132**, 1983–1994 (2005).

42. Norris, A. D., Sundararajan, L., Morgan, D. E., Roberts, Z. J. & Lundquist, E. A. The UNC-6/Netrin receptors UNC-40/DCC and UNC-5 inhibit growth cone filopodial protrusion via UNC-73/Trio, Rac-like GTPases and UNC-33/CRMP. *Development* **141**, 4395–4405 (2014).

43. Steven, R., Zhang, L., Culotti, J. & Pawson, T. The UNC-73/Trio RhoGEF-2 domain is required in separate isoforms for the regulation of pharynx pumping and normal neurotransmission in C. elegans. *Genes Dev.* **19**, 2016–2029 (2005).

44. Alexander, M. *et al.* An UNC-40 pathway directs postsynaptic membrane extension in Caenorhabditis elegans. *Development* **136**, 911–922 (2009).

45. Vanderzalm, P. J. *et al.* C. elegans CARMIL negatively regulates UNC-73/Trio function during neuronal development. *Development* **136**, 1201–1210 (2009).

46. Kishore, R. S. & Sundaram, M. V. ced-10 Rac and mig-2 function redundantly and act with unc-73 trio to control the orientation of vulval cell divisions and migrations in Caenorhabditis elegans. *Dev. Biol.* **241**, 339–348 (2002).

47. Marcus-Gueret, N., Schmidt, K. L. & Stringham, E. G. Distinct cell guidance pathways controlled by the Rac and Rho GEF domains of UNC-73/TRIO in Caenorhabditis elegans. *Genetics* **190**, 129–142 (2012).

48. Williams, S. L. *et al.* Trio’s Rho-specific GEF domain is the missing Galpha q effector in C. elegans. *Genes Dev.* **21**, 2731–2746 (2007).
